# Non‐Membrane Active Peptide Resensitizes MRSA to *β*‐Lactam Antibiotics and Inhibits *S. aureus* Virulence

**DOI:** 10.1002/advs.202416260

**Published:** 2025-02-20

**Authors:** Jingru Shi, Chen Chen, Pan Kong, Feiyu Yu, Qingyan Lv, Zhiqiang Wang, Yuan Liu

**Affiliations:** ^1^ Jiangsu Co‐innovation Center for Prevention and Control of Important Animal Infectious Diseases and Zoonoses College of Veterinary Medicine Yangzhou University Yangzhou 225009 China; ^2^ Joint International Research Laboratory of Agriculture and Agri‐Product Safety the Ministry of Education of China Yangzhou University Yangzhou 225009 China; ^3^ Institute of Comparative Medicine Yangzhou University Yangzhou 225009 China

**Keywords:** antibiotic adjuvant, antibiotic resistance, antimicrobial peptides, MRSA, virulence

## Abstract

Methicillin‐resistant *Staphylococcus aureus* (MRSA) is a serious global health threat due to its high morbidity and mortality rates, creating a dire need for novel therapeutic strategies. Antimicrobial peptides (AMPs), with broad‐spectrum activity and low propensity for resistance development, show promise as effective antibiotic adjuvants to reverse multidrug‐resistance in bacteria. Herein, it is uncovered that a potent and non‐toxic AMP termed GN1 substantially resensitizes MRSA to multiple *β*‐lactam antibiotics at low concentrations. Mechanistic studies indicate that GN1 functions by suppressing both the production and enzymatic activity of MRSA‐associated resistance determinants, including penicillin‐binding protein 2a (PBP2a) and *β*‐lactamase. Additionally, GN1 exhibits a robust anti‐virulence profile by inhibiting MRSA biofilm formation and staphyloxanthin production. Furthermore, GN1 induces bacterial metabolic perturbation, resulting in glutamate accumulation and oxidative damage. Importantly, the combination of GN1 with *β*‐lactam antibiotics effectively mitigates MRSA‐induced infections in the animal infection models. Collectively, these findings suggest that GN1 represents a potent *β*‐lactam adjuvant and anti‐virulence agent, offering a safe and versatile solution to combat MRSA infections.

## Graphical abstract

This work identifies a potent, non‐membrane active, and non‐toxic AMP termed GN1, which substantially resensitizes MRSA to multiple *β*‐lactam antibiotics at low concentrations. GN1 functions by suppressing MRSA‐associated resistance determinants, including penicillin‐binding protein 2a (PBP2a) and *β*‐lactamase. Moreover, GN1 exhibits a robust anti‐virulence profile and induces bacterial metabolic perturbation, thereby combating MRSA‐induced infections.

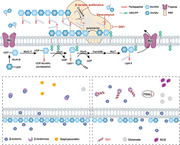

## 1 Introduction

Methicillin‐resistant *Staphylococcus aureus* (MRSA) has emerged as a global health concern since its first appearance in the 1960s, and is a major contributor of both hospital‐acquired and community‐acquired bacterial infections.^[^ ^1^ ^]^ Since it is a leading cause of endocarditis, skin and soft tissue infections, bone and joint infections, MRSA remains a significant clinical threat, accompanied by persistently high morbidity and mortality.^[^ ^2^ ^]^ The discovery of penicillin ushers in the era of antibiotic therapy, but in the 1940s,^[^ ^3^ ^]^ *β*‐lactam resistance soon emerged through the acquisition of the *blaZ* gene, encoding *β*‐lactamase, which was the first identified mechanism of resistance to *β*‐lactam antibiotics. Subsequently, the emergence of MRSA was driven by the acquisition of the staphylococcal cassette chromosome *mec* (SCC*mec*),^[^ ^4^ ^]^ a mobile genetic element that encodes the *mecA* or *mecC* genes. The gene *mecA* encodes penicillin‐binding protein 2a (PBP2a), an enzyme responsible for crosslinking the peptidoglycans in the bacterial cell wall.^[^ ^5^ ^]^ Unlike PBP1, PBP2, PBP3, and PBP4 produced by methicillin‐susceptible *S. aureus* (MSSA), PBP2a produced by MRSA has a poor affinity to *β*‐lactam antibiotics and thus cannot be targeted by these agents. Consequently, PBP2a replaces the biosynthetic function of other PBPs, allowing cell wall synthesis to continue and enabling the bacteria to evade death and lysis.^[^ ^6^ ^]^ Nowadays, MRSA is resistant to most commercially available antibiotics and has become one of the most prevalent clinical “superbugs”.^[^ ^7^ ^]^ Its high morbidity and mortality rates have significantly increased healthcare costs, posing a formidable challenge to public health.

To effectively address MRSA‐induced infectious diseases, the development of novel antimicrobial agents or antibacterial strategies is of paramount urgency. However, over the past decade, the approval of new antibiotics for clinical use has been exceedingly limited, largely due to the substantial technical and financial challenges associated with drug development.^[^ ^8^ ^]^ By contrast, exploring novel antibiotic adjuvants that can enhance the activity of existing antibiotics and extend their lifespan provides a viable and promising alternative strategy.^[^ ^9^ ^]^ Antimicrobial peptides (AMPs), small molecular peptides produced by the host innate immune system,^[^ ^10^ ^]^ are considered one of the most promising alternatives to antibiotics. These peptides are characterized by their high antimicrobial efficiency, broad‐spectrum activity, and low potential for resistance development.^[^ ^11^ ^]^ Additionally, these typically short, cationic and amphipathic molecules can be synthetically designed or optimized based on their amino acid backbone.^[^ ^12^ ^]^ In addition to their direct antimicrobial activity, the synergistic effect of AMPs with existing antibiotics is of growing interest.^[^ ^13^ ^]^ For example, our previous studies have shown that a short linear AMP termed SLAP‐S25 boosted the efficacy of existing antibiotics against multidrug‐resistant (MDR) Gram‐negative bacteria by binding to both lipopolysaccharide (LPS) in the outer membrane and phosphatidylglycerol (PG) in the cytoplasmic membrane.^[^ ^14^ ^]^ Also, we revealed that peptidomimetic 4 (PEP4) was a potent inhibitor of New Delhi Metallo‐*β*‐lactamases (NDMs) and restored meropenem activity^[^ ^15^ ^]^; a surface localized antimicrobial display‐derived cationic peptide SLAY‐P1 specifically antagonized vancomycin resistance in Enterococcus.^[^ ^16^ ^]^ More recently, a non‐toxic AMP, PIS‐A‐1, was shown to kill antibiotic‐tolerant MRSA and exhibited synergism with ampicillin.^[^ ^17^ ^]^ Additionally, the human‐related AMP LL‐37 enhanced the bactericidal effects of azithromycin against MDR Gram‐negative bacteria by increasing outer membrane permeability.^[^ ^18^ ^]^ Despite these ongoing efforts, no peptide compounds have yet been approved as *β*‐lactam adjuvants for the treatment of MRSA infections. Therefore, the identification of potent therapeutic options to combat the escalating antimicrobial resistance crisis remains an urgent and unmet need.

**Figure 1:**
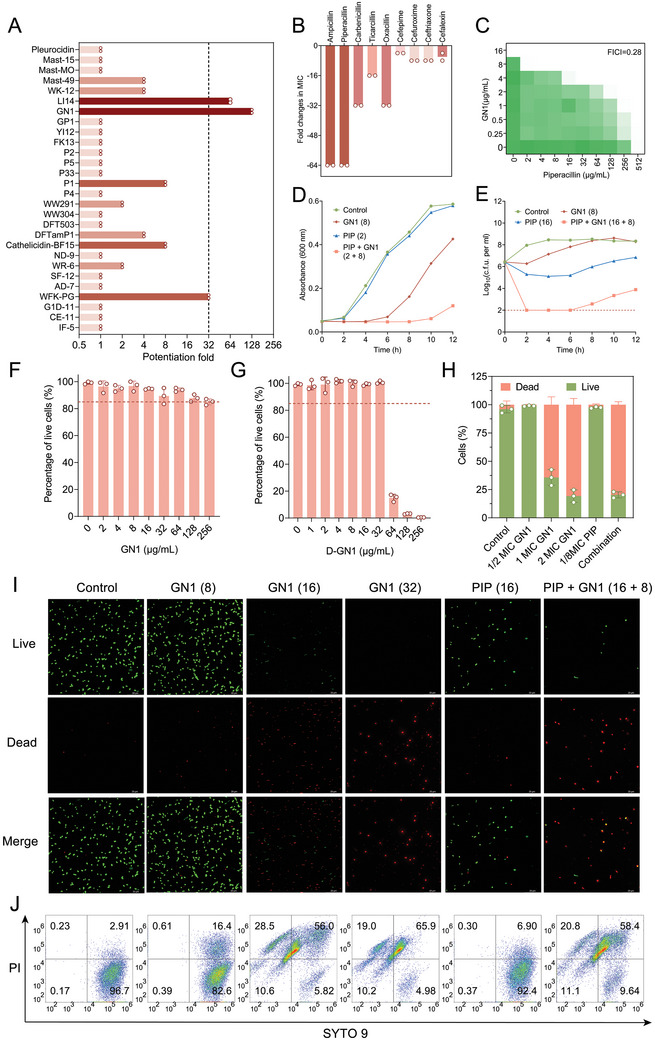
GN1 resensitizes MRSA to *β*‐lactam antibiotics. A) Potentiation of different AMPs to piperacillin (PIP) against MRSA T144. 1/4 MIC AMPs were mixed with bacteria (10^6^ CFU mL^−1^) in MH medium and cultured with diluted PIP in 96‐well plates overnight. B) Fold changes in MIC values of different *β*‐lactams in the presence of GN1 against MRSA T144. C) Checkerboard assay of GN1 and PIP against MRSA T144. OD_600_ nm was measured after 18 h incubation at 37 °C and the fractional inhibitory concentration index (FICI) value was 0.28. D) Growth inhibition of MRSA T144 after incubation with the GN1, PIP alone or their combination. E) Time‐dependent killing of MRSA T144 under the 1/2 MIC GN1, 1/32 MIC PIP alone, or their combination during 12 h. The detection limit of the experiment was 10^2^ CFU mL^−1^. F and G) Cytotoxicity of GN1 (F) and D‐GN1 (G) in macrophage RAW264.7 cells. Cytotoxicity of peptides at different concentrations was evaluated by calculating cell survival rate. H) Fluorescence quantification analysis of the dead and live cells under different treatments using Image J. I) Representative confocal laser scanning microscopy (CLSM) images of MRSA T144 treated with GN1, PIP and their combination, for detecting bacterial viability. Scar bar, 20 µm. J) Live/dead ratios of bacteria, examined by flow cytometry after being treated with GN1, PIP and their combination for 2 h. Viable cells were marked by green fluorescence due to SYTO9 staining, with excitation and emission wavelengths of 485 and 498 nm, respectively. In contrast, dead cells were indicated by red fluorescence due to propidium iodide staining, with excitation and emission wavelengths at 535 and 615 nm, respectively. All experiments were conducted with three biological replicates and data were presented as mean ± SD.

In this study, we conducted a phenotype‐based screening of a group of AMPs (**Figure** **1**; Table S1, Supporting Information) with broad‐spectrum antimicrobial activity to identify new antibiotic potentiators capable of restoring MRSA susceptibility to *β*‐lactam antibiotics. Consequently, we identified a short non‐hemolytic AMP termed GN1, which was designed using training recurrent neural networks (RNN) with data from DBAASP (Database of Antimicrobial Activity and Structure of Peptides).^[^ ^19^ ^]^ GN1 exhibited a potent potentiating effect with *β*‐lactam antibiotics against MRSA, while demonstrating negligible cytotoxicity. Furthermore, we comprehensively assessed the potential of GN1 as novel *β*‐lactams adjuvant both in vitro and in vivo, and elucidated its underlying mechanisms of action.

## 2 Results

### 2.1 GN1 is a Potent *β*‐lactams Adjuvant Against MRSA

To identify AMPs that could restore the efficacy of *β*‐lactams against MRSA, we first evaluated the potentiation of 28 previously reported peptides (at 1/4 MIC) on the activity of piperacillin (PIP) against MRSA T144 using a minimal inhibitory concentration (MIC) assay (Figure S1; Table S1, Supporting Information). As shown in Figure 1A, the potentiation results revealed that the activity of PIP was markedly enhanced in the presence of ten AMPs. Among these, GN1^[^ ^19^ ^]^ and LI14^[^ ^20^ ^]^ were the most potent AMPs, reducing the MIC values of MRSA T144 against PIP by 128‐fold and 64‐fold, respectively. Specifically, GN1 decreased the MIC of PIP from 512 to 4 µg mL^−1^, while LI14 reduced it to 8 µg mL^−1^. Furthermore, we assessed the cytotoxicity of selected AMPs in both RAW264.7 and HEK293T cells. The results indicated that GN1 exhibited the least cytotoxicity within the concentration range of 0–256 µg mL^−1^. In contrast, other AMPs, when used at concentrations ≥ 64 µg mL^−1^, reduced cell viability to below 85% (Figure 1F; Figures S2 and S3, Supporting Information). Given its high potentiation efficacy and low cytotoxicity, GN1 was selected for further investigation.

To investigate whether such potentiation of GN1 to PIP applies to other *β*‐lactams, we evaluated the combination of GN1 with different types of *β*‐lactams, including penicillins, cephalosporins and carbapenems. As demonstrated in Figure 1B; Table S2 (Supporting Information), GN1 fully restored the susceptibility of MRSA T144 to all tested *β*‐lactams, with the highest fold reductions in MIC values observed for ampicillin and PIP. To further examine the synergistic activity of GN1 and PIP against MRSA, we performed checkerboard assays, time‐kill kinetics assays and live/dead bacteria staining. The fractional inhibitory concentration index (FICI) for the GN1‐PIP combination was 0.28 (Figure 1C), and 1/2 MIC GN1 (8 µg mL^−1^) combined with 1/256 MIC PIP (2 µg mL^−1^) effectively inhibited MRSA T144 growth (Figure 1D), which was consistent across other *β*‐lactams (Figure S4, Supporting Information), indicating that GN1 was a lead compound to reverse drug resistance. Meanwhile, the checkerboard assays showed that GN1 also enhanced PIP activity by 32‐fold against other clinical MRSA strains, including MRSA 1518 and MRSA 1530 (Figure S5, Supporting Information). Additionally, the time‐kill kinetics assays revealed that a combination of 1/2 MIC GN1 (8 µg mL^−1^) and 1/32 MIC PIP (16 µg mL^−1^) rapidly eradicated MRSA T144 cells. By contrast, the identical concentrations of GN1 and PIP alone failed to prevent bacterial proliferation (Figure 1E). The live/dead ratios of bacteria were analyzed using flow cytometry and confocal laser scanning microscopy (CLSM), respectively. In the microscopic field of view, bacteria in the control and sub‐MIC of the single drug‐treated group emitted green fluorescence, and those treated with bactericidal concentrations appeared red. Further, combinations of sub‐MIC of the GN1‐PIP group displayed a visual field identical to that of the bactericidal concentration group, characterized predominantly by red light (Figure 1I). Fluorescence quantification analysis confirmed the potent bactericidal effect of the GN1‐PIP combination (Figure 1H). Consistently, flow cytometry analysis also demonstrated a significant reduction in viable bacterial counts in the combination treatment group compared to the control and single‐treatment groups (Figure 1J). Collectively, these results indicate that GN1 remarkably enhances the antibacterial activity of *β*‐lactam antibiotics against MRSA strains.

### 2.2 GN1 is a Safe, Stable, and Non‐Membrane Active AMP

The stability and safety of AMPs are critical prerequisites for their in vivo efficacy. GN1, designed through machine learning to create non‐hemolytic AMPs, has previously been shown to be non‐toxic to human red blood cells.^[^ ^19^ ^]^ Moreover, in our cytotoxicity experiments, GN1 emerged as the safest among the tested peptides, with no significant cell death observed at concentrations up to 256 µg mL^−1^ (Figure 1F; Figure S2 and S3, Supporting Information), suggesting its great safety. To further assess the practicality of GN1 for in vivo applications, we examined its residual antibacterial activity under various conditions, including exposure to different temperatures, pH levels, salt ions, and proteases. Surprisingly, GN1 demonstrated remarkable thermal and pH stability, with its antimicrobial activity remaining unaffected by extreme temperatures and pH conditions. To simulate the in vivo matrix environment, we tested GN1 in culture media containing 10% serum and DMEM. Intriguingly, GN1 retained full activity against MRSA T144 and even exhibited lower MIC values in 10% serum. However, the MIC values of GN1 were elevated to varying degrees in high concentrations of ionic and enzymatic solutions (Table S3, Supporting Information), likely due to the presence of specific enzymatic hydrolysis sites. Given the relative instability of peptides containing L‐amino acids in plasma, we synthesized GN1 with D‐amino acids to enhance its resistance to enzymatic degradation. Consistently, D‐GN1 exhibited superior enzyme stability compared to its L‐type counterpart (Table S4, Supporting Information). However, D‐GN1 was found to be more cytotoxic, resulting in a cell survival rate of less than 20% at 64 µg mL^−1^ (Figure 1G). Therefore, the L‐type GN1 (hereafter referred to as GN1) was selected for further study based on its superior safety profile.

Considering the direct and synergistic antibacterial activity of GN1, we further determined the secondary structure of GN1 in different solvents using circular dichroism (CD) analysis. Lipopolysaccharide (LPS), sodium dodecyl sulfate (SDS) and 50% trifluoroethano (TFEA) were used to simulate bacterial membranes with lipid‐rich, negatively charged and hydrophobic environment, respectively, while PBS served as a blank control. As shown in Figure S6 (Supporting Information), the helix ratio of GN1 increased in LPS, SDS, and TFEA, reaching up to 71.8% and 74.7% in LPS and TFE, respectively, with no strand ratio detected. These results indicated that GN1 may adopt a helical conformation when interacting with membranes. However, unlike the membrane‐active peptide LI14, further experiments showed that GN1 did not affect membrane permeability or potential, and only slightly altered membrane fluidity (Figure S7, Supporting Information). These findings suggested that the antibacterial and synergistic mechanisms of GN1 were not attributed to the canonical membrane‐disruption mode of AMPs. Interestingly, GN1 remarkably reduced the MIC values of *β*‐lactams against MRSA strains but only modestly sensitized drug‐susceptible *S. aureus* (Table S5, Supporting Information), implying that the inhibition of *β*‐lactams resistance mechanism may be associated with the action of GN1.

### 2.3 GN1 Inhibits *β*‐lactam Resistance Determinants of MRSA

**Figure 2:**
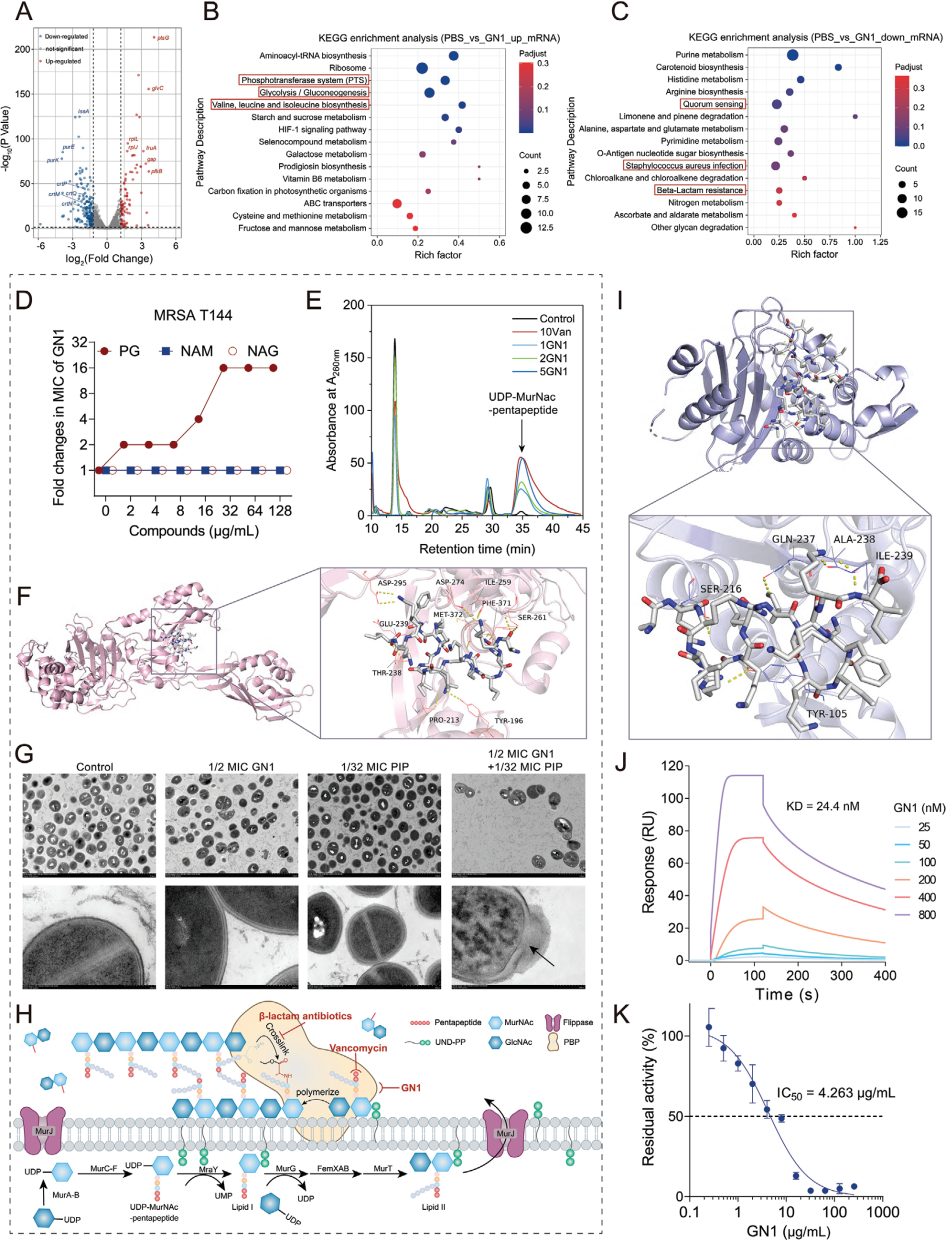
GN1 targets *β*‐lactam resistance determinants of MRSA. A) A volcano map for the distribution of differentially expressed genes (DEGs) of MRSA T144 after exposing to GN1 for 8 h via transcriptome analysis. The x and y axis represent the expression changes and corresponding statistically significant degree, respectively. Each dot represents a specific gene, red dots indicate significantly up‐regulated genes, green dots indicate significantly down‐regulated genes, and gray dots are non‐significantly different genes. An adjusted *P* value < 0.05 (Student's t‐test with Benjamini‐Hochberg false discovery rate adjustment) and |log_2_ Fold change| ≥1 was applied as the cut‐off for significant DEGs. B and C) KEGG (Kyoto Encyclopedia of Genes and Genomes) enrichment analysis of up‐regulated (B) and down‐regulated (C) DEGs. Entries with larger bubbles contain more DEGs. D) Effects of exogenous PG, NAM, and NAG supplementation on the antibacterial activity of GN1. E) The accumulation of UDP‐MurNAC‐pentapeptide, determined by HPLC, vancomycin (Van) was used as a positive control to block cell wall synthesis. F) Molecular docking analysis of the complexes of PBP2a and GN1. In the close‐up view, the hydrogen bonds formed between the compounds and the amino acid residues involved includes Tyr196, Pro213, Thr238, Glu239, Ile259, Ser261, Asp274, Asp295, Phe371 and Met372, which is in the allosteric domain of PBP2a. G) Transmission electron microscopy images of MRSA T144 incubated with PBS (control), 1/2 MIC GN1, 1/32 MIC PIP and their combination. H) Scheme summarizing the synergistic mechanisms of GN1 and PIP in inhibiting bacterial cell wall synthesis. I) Molecular docking analysis of the complexes of *β*‐lactamase and GN1. The amino acid residues of receptors involved includes Tyr105, Ser216, Gln237, Ala238, and Ile239. J) Surface plasmon resonance (SPR) analysis of the binding affinity of GN1 with *β*‐lactamase. K) Dose‐dependent inhibitory effect of GN1 on *β*‐lactamase activity. IC_50_, half‐maximal inhibitory concentration. Experiments were conducted with three biological replicates and data were presented as mean ± SD.

To elucidate the mechanisms underlying the potentiation of GN1 to *β*‐lactams, we conducted RNA‐sequencing analysis to identify the differentially expressed genes (DEGs) in MRSA T144 treated with GN1. As shown in **Figure** **2**A, genes with at least a twofold change in expression compared to the control group were selected. The volcano plot revealed a total of 374 DEGs, comprising 121 upregulated and 253 downregulated genes. KEGG enrichment analysis further indicated that these up‐regulated DEGs were involved in various metabolic activities and metabolism pathways (Figure 2B). Specifically, the downregulated DEGs were associated with pathways such as quorum sensing, *S. aureus* infection, and *β*‐lactamase resistance (Figure 2C). Notably, GN1 significantly downregulated the expression levels of *β*‐lactam resistance‐related genes, including the *mecA* gene (encoding PBP2a, downregulated by 1.78‐fold) and the *blaZ* gene (encoding *β*‐lactamase, downregulated by 2.82‐fold) (Figure S8, Supporting Information).

On the basis of the transcriptome results above, we first investigate whether GN1 affects the cell wall synthesis of MRSA. To this end, we assessed the impact of exogenous addition of peptidoglycan (PG) and its structural components, including acetylglucosamine (NAG) and acetylmuramic acid (NAM), on the antibacterial activity of GN1. As shown in Figure 2D, the results showed that the MIC values of GN1 gradually increased with increasing PG concentration, while NAG and NAM had no effect on the activity of GN1. High‐performance liquid chromatography (HPLC) analysis further revealed that the accumulation of UDP‐MurNAC‐pentapeptide, a soluble peptidoglycan precursor, increased with GN1 concentrations (Figure 2E), indicating that GN1 hindered cell wall synthesis. Furthermore, we investigated the binding of PBP2a with GN1 via molecular docking analysis. SiteMap identified five potential binding sites on PBP2a (PDB ID: 5M18), with site 1 located at the allosteric site and site 2 at the active site (Figure S9, Supporting Information).^[^ ^21^ ^]^ The activation of the allosteric site would change the initially closed active center into an accessible state for *β*‐lactam antibiotics,^[^ ^21b^ ^]^ thus inhibiting the synthesis of peptidoglycan and cell wall finally. AutodockVina results confirmed that GN1 could bind to the allosteric site of protein PBP2a (PDB ID: 5M18) (Figure 2F), with the lowest binding energy of −5.3 kcal mol^−1^ (Table S6, Supporting Information). Transmission electron microscopy (TEM) further demonstrated that untreated or single‐treated bacteria had well‐defined cell walls, while the combination of GN1 and PIP disrupted cell wall integrity (Figure 2G). These findings support the notion that GN1 restores *β*‐lactam efficacy against MRSA by interfering with peptidoglycan synthesis (Figure 2H).

In addition to PBP2a, *β*‐lactamase encoded by the *blaZ* gene also confers high‐level resistance to *β*‐lactams by destroying their pharmacodynamic groups.^[^ ^22^ ^]^ Notably, the expression of *blaZ* was also reduced by 2.82‐fold upon treatment with GN1 compared to that in the control group (Figure S8, Supporting Information). Moreover, we analyzed whether GN1 can directly bind to *β*‐lactamase. SiteMap identified the first four potential binding sites on the *β*‐lactamase (PDB:1ALQ) (Figure S9B), and the molecular docking results showed that GN1 could directly bind to site 2 of *β*‐lactamase (Figure 2I; Table S7, Supporting Information). To further verify it, we determined the binding affinity of GN1 with *β*‐lactamase via surface plasmon resonance (SPR) analysis. The results indicated that GN1 can directly interact with *β*‐lactamase, with a binding affinity characterized by an equilibrium dissociation constant (KD) of 2.44 × 10^−8^ M (Figure 2J). To support this, the enzymatic activity of *β*‐lactamase in the presence of GN1 was also evaluated using nitrocefin. As expected, the activity of *β*‐lactamase was dose‐dependently suppressed by GN1, with an IC_50_ of 4.263 µg mL^−1^ (Figure 2K). Taken together, our results indicate that GN1 restore the susceptibility of MRSA to *β*‐lactams by simultaneously inhibiting the activity of resistance enzymes and downregulating the expression levels of related resistance genes.

### 2.4 GN1 Attenuates *S. aureus* Virulence by Reducing the Production of Biofilms and Staphyloxanthin

**Figure 3:**
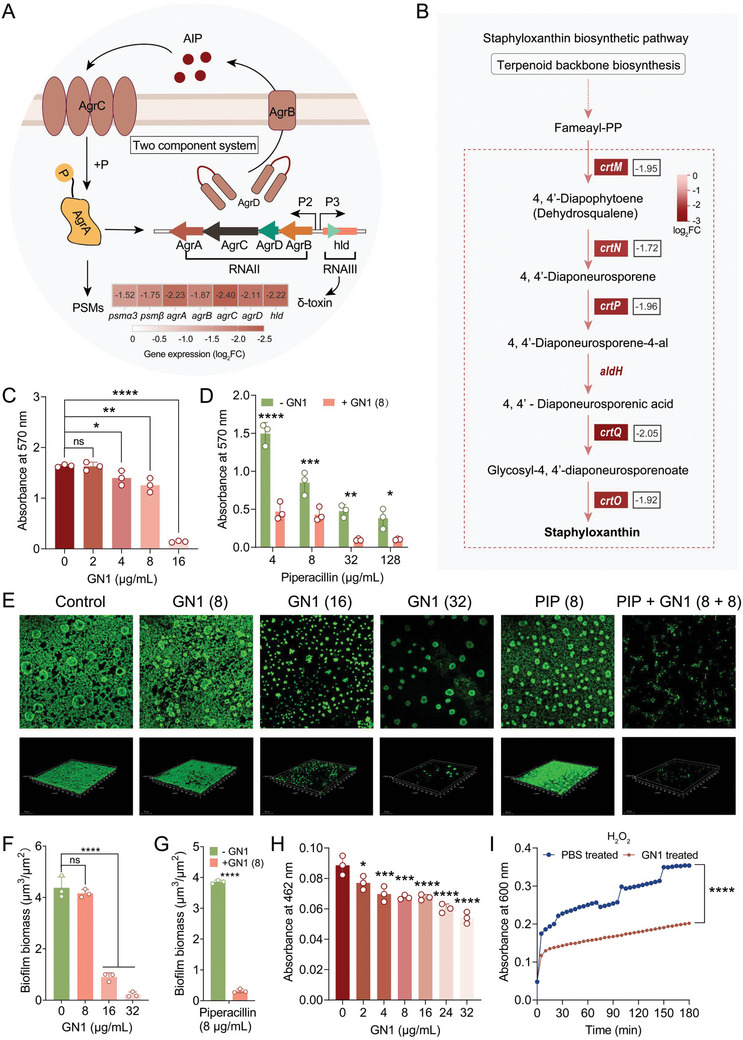
GN1 displays anti‐virulence properties against MRSA. A) Phenol‐soluble modulins (PSMs) regulation pathway. B) Schematic of staphyloxanthin biosynthesis pathway. The genes inducing staphyloxanthin biosynthesis were down‐regulated to varying degrees in MRSA T144 exposed to GN1. C) Biofilm formation of MRSA T144 cells after exposure to varying concentrations of GN1. D) Biofilm formation of MRSA T144 cells under different concentrations of PIP or in combination with GN1(8 µg mL^−1^). Biofilms was stained with crystal violet and the mass was measured at the absorbance of 570 nm. E) Representative confocal laser scanning microscopy (CLSM) images of MRSA T144 biofilm formation treated with GN1, PIP alone, and their combination. Scar bar, 20 µm. F,G) The quantification of biomass of corresponding biofilm formed by MRSA T144 treated with different concentrations of GN1, PIP alone and their combination, related to Figure 3E. H) Effect of GN1 on the staphyloxanthin biosynthesis of MRSA T144. Bacteria were incubated with different concentrations of GN1 at 37 °C for 24 h, and then extracted with methanol and the absorbance value at 462 nm was determined accordingly. I) Effect of GN1 on the susceptibility of MRSA T144 to H_2_O_2_. After exposed to PBS or GN1, the bacteria were incubated with H_2_O_2_ and the growth status at OD_600_ was monitored. All experiments were conducted with three biological replicates and data were presented as mean ± SD. Statistical significance was analyzed by ordinary one‐way ANOVA or two‐way ANOVA (*P < 0.05, **P < 0.01, ***P < 0.001, ****P < 0.0001). ns, not significant.

Another significant pathway inhibited by GN1 is the quorum sensing (QS) system, which operates through the accessory gene regulator (agr) system. This two‐component regulatory system governs the production of numerous virulence factors in *S. aureus* (Figure 2C).^[^ ^23^ ^]^ Our RNA‐sequencing analysis revealed that GN1 downregulated genes encoding phenol‐soluble modulins (PSMs), key virulence determinants in MRSA, including *psmα3* and *psmβ*. PSMs play multiple roles in staphylococcal pathogenesis, including lysing red and white blood cells, stimulating inflammatory responses, and contributing to biofilm development and the dissemination of biofilm‐associated infections (**Figure** **3**A). Considering the expression of PSM genes is positively and strictly controlled by the *agr*,^[^ ^24^ ^]^ we reasoned that GN1 may inhibit agr‐QS system activity, highlighting its potential as an anti‐virulence agent (Figure 2C).

The *agr* phenotype also influences several aspects of biofilm behavior, including surface attachment, biofilm dispersal, and the chronicity of biofilm‐associated infections. Additionally, MgrA regulates PSM production to modulate biofilm formation and detachment in *S. aureus*.^[^ ^25^ ^]^ Biofilm is an important factor enabling MRSA to resist adverse environments and spread virulence. Therefore, we evaluated the anti‐biofilm activity of GN1, both alone and in combination with PIP. As shown in Figure 3C, sub‐MIC concentrations of GN1 dose‐dependently reduced MRSA adhesion and prevented biofilm formation. Furthermore, GN1 enhanced the inhibitory effects of PIP and other *β*‐lactams on biofilm development (Figure 3D; Figure S10, Supporting Information). Consistently, live/dead bacterial staining demonstrated that the combination of sub‐MIC GN1 and PIP effectively reduced biofilm viability (Figure 3E). Specifically, biofilm biomass formed by MRSA T144 in the presence of GN1‐PIP was significantly lower than that in the single treatment group, as analyzed using ImageJ and COMSTAT software^[^ ^26^ ^]^ (Figure 3F,G).

Staphyloxanthin, a golden carotenoid pigment and a crucial virulence factor of *S. aureus*, protects the bacteria from oxidative killing by the host.^[^ ^27^ ^]^ KEGG pathway enrichment analysis revealed that GN1 significantly downregulated genes associated with carotenoid biosynthesis (Figure 2C). The proposed biosynthetic pathway for staphyloxanthin involves six enzymes: 4,4′‐diapophytoene synthase (CrtM), 4,4′‐diapophytoene desaturase (CrtN), 4,4′‐diaponeurosporene oxidase (CrtP), 4,4′‐diaponeurosporen‐aldehyde dehydrogenase (AldH), glycosyltransferase (CrtQ) and acyltransferase (CrtO) (Figure 3B). The expression of all these genes was substantially downregulated by GN1 (Figure 2C). Consistent with the RNA‐sequencing results, we observed that GN1 inhibited staphyloxanthin biosynthesis in a concentration‐dependent manner (Figure 3H). Given that this pigment protects S. aureus from host oxidant killing,^[^ ^27^ ^]^ we examined whether GN1 enhances the susceptibility of MRSA T144 to hydrogen peroxide (H_2_O_2_)‐mediated lethality. The growth curves of MRSA T144 treated with H_2_O_2_ after pre‐exposure to PBS or GN1 revealed that GN1 pre‐treatment increased MRSA susceptibility to H_2_O_2_ treatment (Figure 3I). Together, these results demonstrate that GN1 acts as a potential anti‐virulence agent by reducing biofilms formation and staphyloxanthin production in *S. aureus*, thereby enhancing bacterial susceptibility to oxidative stress.

### 2.5 GN1 Triggers Metabolic Perturbation and Oxidative Damage

**Figure 4:**
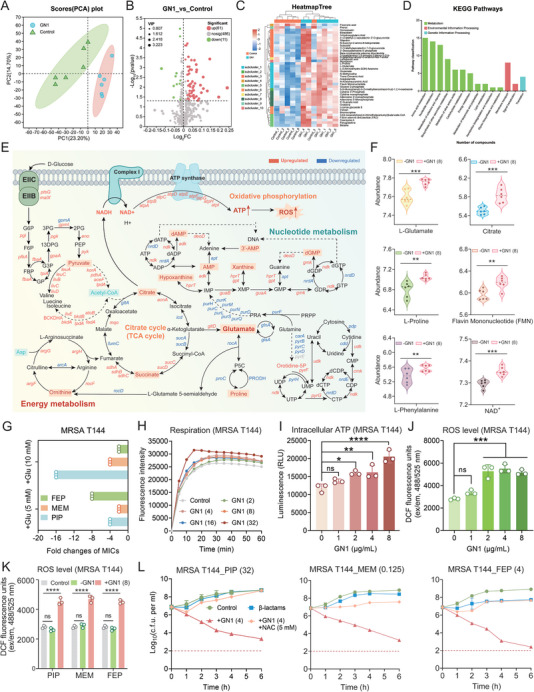
GN1 induces bacterial metabolic perturbation. A) Principal Component Analysis (PCA) of samples treated with GN1. The degree of aggregation and dispersion between samples is represented by the distance of each coordinate point; the closer the distance, the more similar the samples are, and the longer the distance, the more different the samples are. B) A volcano map for the distribution of Differential metabolites of MRSA T144 after exposing to GN1 for 8 h. The x and y axis represent the fold change value of the expression changes and corresponding statistically significant degree, respectively. Each dot represents a specific metabolite; red dots indicate significantly up‐regulated metabolites, green dots indicate significantly down‐regulated metabolite, and gray dots are non‐significantly different metabolites. C) Metabolite clustering heat map showing the trend of differential metabolites in different groups. The name of the metabolite is shown on the right, and the dendrogram of metabolite clustering is shown on the left. The closer the two metabolite branches are, the more similar their expression levels are. The closer the two samples' branches are, the closer all of the metabolites' expression patterns are in the two samples, and the closer the two samples' metabolite expression trends are. D) KEGG pathway, which processes metabolic pathway information in which metabolites are involved. The KEGG metabolic pathway's secondary classification is represented by the ordinate, while the number of metabolites annotated to the route is shown by the abscissa. E and F) Combined transcriptomics and metabolomics analysis of MRSA T144 after exposure to GN1 or PBS. Most of the genes and metabolites related to energy metabolism shown were up‐regulated. Phosphotransferase system (PTS) and glycolysis/gluconeogenesis were significantly activated. G) Fold changes in MIC values of various *β*‐lactams in the presence of glutamate against MRSA T144. H–K) Bacterial respiration level (H), intracellular ATP (I) and ROS levels (J and K) of MRSA T144 treated with different concentrations of GN1 or in combination with *β*‐lactam antibiotics. L) The impact of ROS scavenger *N*‐acetyl‐L‐cysteine (NAC) supplementaion on the synergistic activity of GN1 and various *β*‐lactam antibiotics against MRSA T144. All experiments were conducted with three biological replicates and data were presented as mean ± SD. Statistical significance was analyzed by ordinary one‐way or two‐way ANOVA (*P < 0.05, **P < 0.01, ***P < 0.001, ****P < 0.0001). ns, not significant.

Antibiotic‐mediated bacterial death is a complex process involving multiple factors beyond the initial drug‐target interaction. The metabolic status of bacteria influences their susceptibility to antibiotics, and metabolic byproducts generated in response to antibiotic stress can contribute to cellular damage and death.^[^ ^28^ ^]^ Given that bacterial central metabolic pathways are closely linked to antibiotic activity and resistance,^[^ ^29^ ^]^ we performed metabolomics analysis to further explore the mechanisms underlying the broad‐spectrum potentiation of GN1 to *β*‐lactams (**Figure** **4**A). Compared to the untreated group, the results revealed significant variations in 72 metabolites upon GN1 treatment, with 61 metabolites upregulated and only 11 downregulated (Figure 4B). KEGG enrichment analysis highlighted metabolites primarily involved in amino acid metabolism (Figure 4C,D). Integrating these results with transcriptomic and RT‐qPCR analyses, we found that genes and metabolites related to energy metabolism were predominantly upregulated following GN1 treatment. These pathways included the phosphotransferase system (PTS), pyruvate cycle, ornithine cycle, tricarboxylic acid (TCA) cycle, and ATP synthesis, which collectively provide sufficient substrates and ATP for glutamate synthesis. Conversely, analysis of glutamate downstream products showed significant downregulation of glutamate decomposition pathways, leading to intracellular glutamate accumulation (Figure 4E,F; Figure S11, Supporting Information). Based on these findings, we hypothesized that glutamate accumulation may play a crucial role in the synergistic activity between GN1 and *β*‐lactam antibiotics.

To test this hypothesis, we assessed the susceptibility of *β*‐lactam antibiotics in the presence of glutamate. Interestingly, the addition of glutamate reduced the MIC values of MRSA T144 against three different *β*‐lactam antibiotics (2‐fold to 16‐fold), indicating that glutamate enhances the antibacterial activity of *β*‐lactams (Figure 4G). Further quantitative analysis revealed that exogenous glutamate significantly increased the intracellular accumulation of PIP (Figure S12, Supporting Information). Thus, we reasoned that GN1 treatment could induce a robust metabolic response that drives glutamate accumulation, which in turn amplifies the intracellular uptake of *β*‐lactam antibiotics, thereby enhancing bacterial sensitivity to these agents.

The enhanced metabolic response is necessarily accompanied by increased respiration, leading to the accumulation of toxic byproducts. Upon GN1 treatment, bacterial respiration levels were upregulated (Figure 4H), and intracellular ATP levels increased with rising GN1 concentrations (Figure 4I). In addition, the production of reactive oxygen species (ROS) was significantly elevated in bacteria treated with increasing concentrations of GN1 (Figure 4J). Furthermore, when GN1 was combined with *β*‐lactams, ROS production was higher compared to treatments with antibiotics alone (Figure 4K). Further bactericidal curve analysis showed that the synergistic bactericidal activity of the combination was significantly attenuated upon addition of the ROS scavenger *N*‐acety‐L‐cysteine (NAC) (Figure 4L), suggesting a direct correlation between ROS overproduction and the potentiating effect of GN1. Together, our results demonstrate that the enhanced metabolic state induced by GN1 plays a critical role in its synergistic activity with *β*‐lactam antibiotics.

### 2.6 GN1 Effectively Rescues *β*‐lactams Efficacy In Vivo

**Figure 5:**
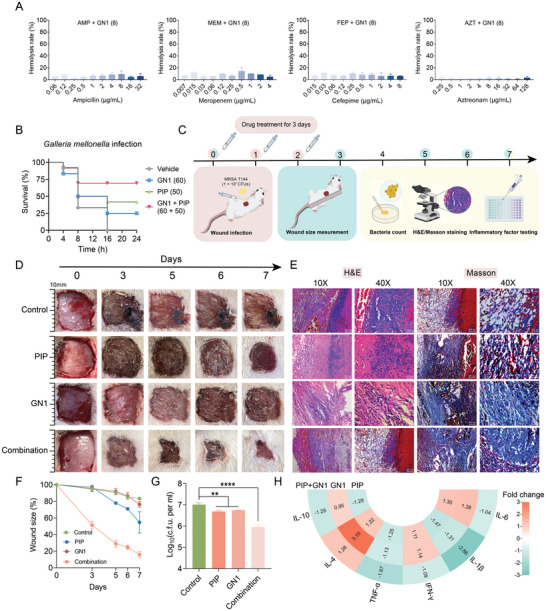
GN1 improves the effectiveness of piperacillin in MRSA‐induced animal infection models. A) Hemolytic activity of GN1 (8 µg mL^−1^) plus various concentrations of *β*‐lactam antibiotics for on mammalian RBCs. B) Survival rate of *Galleria mellonella* larvae (n =12 per group) infected by MRSA T144 after drug treatments. C) Model of timeline and operation of murine skin wound infections (n = 4 per group). The mouse wounds were infected with 1.0 × 10^7^ CFUs of MRSA T144, and then treated with PBS, GN1 (5 mg kg^−1^), PIP (25 mg kg^−1^) and the drug combination after 1 h for 3 days. D) Representative photographs of wounds infected with 1.0 × 10^7^ CFUs MRSA T144, under the treatments of GN1, PIP and their combination. Photographs were taken on days 0, 3, 5, 6, 7 to record the wound condition. E) Histopathological evaluation of wound tissues on day 7 using H&E and Masson staining. F) Percentage of wound size measured at the 3^rd^, 5^th^, 6^th^, and 7^th^ day. G) Bacterial loads in the wound tissues after drug treatments, determined by quantitative culturing from day 7 of the infection sites. All experiments were conducted with three biological replicates and data were presented as mean ± SD. Statistical significance was analyzed by ordinary one‐way ANOVA (**P < 0.01 and ****P < 0.0001). H) Fold changes of the expression of cytokines in mice wounds after treatment with GN1, PIP and their combination.

*S. aureus* can enter the bloodstream or underlying tissues and cause infection when the epidermal and mucosal barriers are compromised. To demonstrate the safety and therapeutic potential of GN1 and PIP combination for combating MRSA infections in vivo, we evaluated the hemolytic effect of drug combinations on mammalian red blood cells (RBCs) and assessed the efficacy of GN1‐PIP in two MRSA‐induced animal infection models. The hemolysis assay showed that 1/2 MIC (8 µg mL^−1^) GN1 combined with various concentrations of *β*‐lactam antibiotics did not cause hemolysis to mammalian erythrocytes (**Figure** **5**A). Using the *Galleria mellonella* infection model, we demonstrated that compared to GN1 or PIP administered alone, the combination treatment improved larval survival rates within 24 h (Figure 5B). In the rat skin wound infection model, the wounded skin was infected with MRSA T144 and treated with GN1, PIP alone and their combination at 1 h post‐infection (Figure 5C). Compared to the monotherapy groups, the wound size treated with GN1‐PIP combination was significantly reduced by 35%‐50% within 7 days (Figure 5D,F). Histological analysis using H&E and Masson staining revealed a significant decrease in inflammatory cell infiltration and an increase in collagen deposition in wounds treated with the GN1‐PIP combination (Figure 5E). Additionally, the number of bacterial colony‐forming units (CFUs) in wounds treated with GN1‐PIP was significantly lower than that in the control and monotherapy groups (Figure 5G). Consistent with the improved tissue healing, the GN1‐PIP combination also suppressed the production of pro‐inflammatory cytokines (TNF‐α, IFN‐γ, IL‐1*β*, and IL‐6) (Figure 5H), suggesting potential immunomodulatory properties of GN1. These results together demonstrate that GN1 rescues the effectiveness of *β*‐lactams against MRSA‐elicited infections.

## 3 Discussion

MDR bacterial pathogens compromise current antibiotic therapy and pose a serious threat to public health.^[^ ^30^ ^]^ Among these, MRSA has demonstrated an extraordinary capacity for evolution and widespread dissemination, imposing substantial medical and economic burdens since its initial recognition. Alarmingly, some MRSA strains have even developed resistance to vancomycin, a last‐resort antibiotic against serious infection caused by Gram‐positive bacteria.^[^ ^31^ ^]^ The MDR phenotype of MRSA to most antibiotics, coupled with the high difficulty in developing new drugs, underscores the urgent need to explore alternative therapeutic strategies. Drug combination approaches offer a promising strategy to combat MRSA infections. Accumulating evidence indicates that AMPs can serve as potential antibiotic adjuvants to reverse resistance in MDR pathogens.^[^ ^14^, ^17^, ^32^ ^]^ In this study, we identified a non‐membrane active, stable and safe AMP named GN1 as a novel *β*‐lactams adjuvant and anti‐virulence agent. Notably, GN1 dramatically potentiated the antibacterial activity of *β*‐lactams against MRSA strains both in vitro and in murine infection models.

Regarding the mechanism underlying the potentiation of GN1 to *β*‐lactams, our findings demonstrated that GN1 differs from canonical AMPs, which typically increase cell membrane permeability to facilitate antibiotic entry into bacterial cells and access to intracellular targets, thereby enhancing bacterial killing.^[^ ^13^ ^]^ By contrast, GN1 did not disrupt bacterial membrane permeability or potential. Instead, its potentiating effects are attributed to its ability to overcome MRSA‐associated resistance mechanisms. MRSA resistance to *β*‐lactams is generally mediated by *β*‐lactamase production (encoded by *blaZ*) and the expression of penicillin‐binding protein 2a (PBP2a, encoded by *mecA*).^[^ ^33^ ^]^ For instance, the effectiveness of *β*‐lactamase inhibitors, such as clavulanic acid,^[^ ^34^ ^]^ has been clinically proven for decades. Intriguingly, our findings revealed that GN1 not only downregulated the expression of resistance genes, but also targeted the pathway of peptidoglycan synthesis and suppressed the activity of PBP2a, thereby inhibiting cell wall synthesis in conjunction with *β*‐lactam antibiotics. Furthermore, GN1 displayed strong binding affinity with *β*‐lactamases and antagonized its hydrolytic activity toward *β*‐lactam antibiotics. These multifaceted inhibitory effects of GN1 on the resistance determinants of MRSA account for its excellent synergistic activity with *β*‐lactams against MRSA.

Accumulating evidence demonstrates that the potency of bactericidal antibiotics is directly linked to the metabolic state of bacteria.^[^ ^35^ ^]^ The transcriptional response to these bactericidal agents often involves the upregulation of genes associated with central metabolism and respiration.^[^ ^36^ ^]^ Metabolomic profiling of *Mycobacterium tuberculosis* treated with a range of bactericidal agents has also revealed consistent patterns in the restructuring of central metabolic pathways.^[^ ^37^ ^]^ In terms of cellular metabolic status, the efficacy of bactericidal antibiotic therapy correlates with carbon flow through the TCA cycle,^[^ ^38^ ^]^ and environmental factors like molecular oxygen availability also play a role in antibiotic killing.^[^ ^28^, ^39^ ^]^ In our study, we found that GN1 not only inhibited resistance mechanisms but also disrupted metabolic pathways. Transcriptome and metabolomics analyses revealed that GN1 treatment interfered with the MRSA energy metabolism pathway, including pyruvate cycle, TCA cycle, and electron transport chain. This interference is accompanied by activation of bacterial respiration and a significant increase in intracellular ATP and ROS production. Consistent with previous findings, metabolically active bacteria are more susceptible to bactericidal antibiotics.^[^ ^35^ ^]^ Additionally, glutamate, a product of bacterial energy metabolism, is significantly upregulated in response to GN1 treatment. Also, exogenous glutamate supplementation enhanced the antibacterial activity of *β*‐lactam antibiotics, in line with a recent study showing that exogenous glutamine can potentiate the killing efficacy of *β*‐lactams, aminoglycosides, quinolones, and tetracyclines against uropathogenic *E. coli*.^[^ ^40^ ^]^ However, the precise mechanisms and role of glutamate in the synergistic effect between GN1 and *β*‐lactam antibiotics warrant further exploration.

Bacterial virulence factors are capable of damaging the host and evading the immune system, thereby causing infections. In *S. aureus*, key regulatory systems, including the transcriptional regulators of the quorum sensing system Agr, the two‐component regulatory system SaeRS and the Sar family proteins, control these virulence factors and help the bacteria resist oxidative stress and immune attacks.^[^ ^41^ ^]^ Additionally, the synthesis of the protective pigment staphyloxanthin is crucial for *S. aureus* survival and pathogenesis.^[^ ^42^ ^]^ Alongside the development of new antimicrobial agents, anti‐virulence strategy has emerged as a highly promising approach to counteract bacteria‐mediated diseases.^[^ ^43^ ^]^ Unlike traditional antimicrobial approach aimed at killing bacteria or inhibiting their growth, anti‐virulence strategies exert less selective pressure for resistance development.^[^ ^44^ ^]^ Biofilms, a critical virulence factor in the pathogenesis of opportunistic bacteria, exacerbate persistent infections in patients and exhibit increased resistance to antimicrobial agents.^[^ ^45^ ^]^ Our findings revealed that GN1 significantly inhibited biofilm formation and pigment synthesis, positioning it as a potent anti‐virulence compound for the treatment of *S. aureus* infections. This dual action of GN1 suggests a novel therapeutic avenue that could complement existing antimicrobial therapies and mitigate the impact of biofilm‐associated infections.

In summary, we demonstrate that a versatile peptide GN1, coupled with robust synergistic effect, anti‐virulence properties, and satisfied safety profiles, represents a promising *β*‐lactams adjuvant to tackle the recalcitrant infection caused by MRSA. Meanwhile, the underlying mechanisms of GN1 provide valuable insights for the future development of novel antibiotic adjuvants. Further clinical trials are warranted to assess the practical application and therapeutic potential of GN1 in clinical settings.

## 4 Experimental Section

### Peptide Synthesis and Validation

All peptides employed in this study were synthesized using solid‐phase techniques by GL Biochem (Shanghai, China). Helical wheels of AMPs (Figure S1, Supporting Information) were presented using HeliQuest analysis (http://heliquest.ipmc.cnrs.fr/cgi‐bin/ComputParamsV2.py).

### Bacterial Strains and Growth Conditions

All MRSA strains including MRSA T144, MRSA 1518 and MRSA 1530 were stored in nutrient broth supplemented with 20% glycerol at −80 °C. Unless otherwise noted, LB broth or LB agar was used for the normal growth of all bacteria.

### Animal Studies and Ethical Statement

ICR male rats (≈250 g) were obtained from the Comparative Medicine Center of Yangzhou University. Prior to infection studies, all the rats were fed under standardized environmental conditions for one week. In this study, all animal experiments were carried out according to the guidelines of Jiangsu Laboratory Animal Welfare and Ethical of Jiangsu Administrative Committee of Laboratory Animals (SYXK‐2022‐0044). The laboratory animal usage license number is SCXK‐2022‐0009, certified by the Jiangsu Association for Science and Technology.

### Antibacterial and Bactericidal Activity Tests—Minimal Inhibitory Concentration (MIC) Assay

MICs determination of drugs was based on the standard microdilution method as described previously.^[^ ^46^ ^]^ Briefly, compounds were twofold serial dilution with MHB. Next, AMPs were added to the log‐phase bacteria suspensions (1 × 10^6^ CFUs mL^−1^) and then mixed with compounds in a 96‐well microliter plate (Corning, NY, USA). Plates were incubated at 37 °C overnight (16–18 h), and the MIC values of the compounds were defined as the lowest concentration of drugs that no visible bacterial growth.

### Checkerboard Assay

Synergistic activities of the combination were tested by checkboard assays as described previously.^[^ ^47^ ^]^ Briefly, 100 µL of MHB was dispensed into a 96‐well microliter plate. Bacterial culture was grown to log phase and diluted to 1 × 10^6^ CFUs mL^−1^. After 18 h incubation at 37 °C, the absorbance of each well at 600 nm was measured by a Microplate reader (Tecan Infinite E Plex). The fractional inhibitory concentrations index (FICI) was calculated accordingly. FICI ≤ 0.5 demonstrates synergy.

### Time‐Killing Assay

The kill kinetics of GN1 combined with *β*‐lactams against MRSA T144 was determined as previously described.^[^ ^48^ ^]^ Briefly, overnight bacteria culture was diluted 1:1000 into LB and incubated for 4 h at 37 °C with 200 rpm. Then, the culture was diluted into LB at 1:1,00 and treated by either PBS, *β*‐lactams, GN1, or *β*‐lactams in combination with GN1. At the time points 0, 2, 4, 6, 8, 10, and 12 h, 100 µL bacteria culture was removed and resuspended in PBS and the serial dilutions were spotted on LB agar. After incubation overnight at 37 °C, the colony counts were calculated.

### Salt and Serum Stability

MRSA T144 was incubated with GN1 supplemented with 10 mM Na^+^, K^+^, Mg^2+^, Fe^3+^, 10% Dulbecco's Modified Eagle Medium (DMEM), or fetal bovine serum (FBS) for 16–18 h. The stability of GN1 was assessed by determining the changes of MIC values.

### Flow Cytometry

The viability and quantity of the bacterial populations were examined using flow cytometry as previously described.^[^ ^20^ ^]^ Briefly, 10^6^ CFUs mL^−1^ bacteria suspension was incubated with GN1, PIP, or their combination for 2 h at 37 °C. Then, PI (5 mM, 3 µL) and SYTO 9 (0.835 mM, 3 µL) were added to the mixture of bacteria and drugs and incubated for 15 min at room temperature in dark. The samples were detected using a flow cytometer with FITC and PI channels (Beckman Coulter, USA). Data were analyzed using FlowJo software (TreeStar, USA).

### Thermal, pH and Proteolytic Stability

GN1 was preincubated at different temperatures (20−121 °C), pH (2−12), and proteases (pepsin, trypsin, and papain, final concentration 1 mg mL^−1^) for 1 h.^[^ ^49^ ^]^ Then, all samples were readjusted to pH = 7.2 to determine the residual antibacterial activity by MIC tests. After proteases incubation, the remaining protease was precipitated using acetonitrile and removed by centrifugation at 3000 g, followed by a MIC test.

### Circular Dichroism (CD) Spectrum Assay

GN1 was respectively dissolved in PBS (0.01 m, pH 7.2), LPS (50 µm), SDS (50 mm), and 50% TFEA, with a final concentration of 0.1 mg mL^−1^.^[^ ^50^ ^]^ CD values were measured at 25 °C with a spectrum of 190–300 nm by a J‐810 spectropolarimeter (Jasco, Tokyo, Japan).

### Confocal Laser Scanning Microscopy (CLSM) Analysis

Cells exposed to PBS, GN1, PIP or their combination were imaged by CLSM.^[^ ^51^ ^]^ Briefly, bacterial cultures were inoculated in sterile 24‐well plates containing tested compounds. Sterile Ti6Al4 V disks were then immersed into the medium as a substratum for biofilm growth and incubated at 37 °C for 24 h. Planktonic cells were removed by rinsing three times with PBS, and bacterial viability was determined using PI (5 mm, 3 µL) and SYTO 9 (0.835 mm, 3 µL). After incubating for 15 min in the dark, the stained disks were observed using a CLSM microscope (Leica TCS SP8 STED; Heidelberg, Germany).

### Transcriptomic Analysis

MRSA T144 was grown in MHB to the early‐exponential phase and treated with GN1 (1/2 MIC) for 8 h.^[^ ^20^ ^]^ After incubation, cells were harvested, and the total RNA of samples was extracted using Bacteria RNA Extraction Kit (Vazyme, Nanjing, China), and quantified by using a Nanodrop spectrophotometer (Thermo Scientific, MA, USA), sequenced by the Illumina Hiseq 2000 system (Majorbio, Shanghai, China).

### Molecular Docking

Molecular docking site identification was performed with Schrodinger docking suits (Schrödinger Maestro, New York) using a virtual screening workflow. The chemical structure of GN1 was prepared using ChemDraw software and the crystal structures of the protein was obtained from the Protein Data Bank (PDB). Both ligands and receptors were preprocessed by Pymol software. Docking was subsequently performed using the AutoDock Vina function of AutoDockTools software. The best‐generated models were chosen for interaction analysis and figures for the structure models were generated using Pymol.

### Transmission Electron Microscopy (TEM)

The cell wall structure changes in the bacteria after treatment were examined using a Transmission electron microscope (TEM).^[^ ^52^ ^]^ Briefly, log‐phased bacterial cultures were treated with GN1, PIP, the combination of them and PBS (control) for 8 h at 37 °C. Next, the bacterial suspension was centrifuged and resuspended in 1 mL of a fixative solution. Then, fixed bacteria were washed three times with 0.1 m cacodylate buffer and post‐fixed with 1% osmium tetroxide for 1 h. The bacteria were washed three times with water and further dehydrated with an alcohol gradient series (10 min each: 50%, 60%, 70%, 80%, 90%, and 100%). Subsequently, the samples were infiltrated with Epon resin and polymerized at 75 °C for 48 h. Ultrathin sections were cut with a diamond knife, picked up on a copper grid, and stained with lead citrate. Micrographs of the cells were observed using a JEM 1011 TEM (JEOL, Tokyo, Japan).

### Surface Plasmon Resonance (SPR) Analysis

The CM5 sensor chip was activated by mixing 400 mm EDC and 100 mm NHS immediately prior to injection. The activation was performed for 420 s at a flow rate of 10 µL min^−1^. *β*‐lactamase was diluted to 200 µg mL^−1^ in immobilization buffer and injected into the sample channel at a flow rate of 10 µL min^−1^. GN1 was prepared at seven different concentrations (800, 400, 200, 100, 50, and 25 µm) using the same analyte buffer. GN1 was injected into channels at a flow rate of 30 µL min^−1^, with an association phase of 120 s followed by a dissociation phase of 300 s. Both association and dissociation processes were conducted in the analyte buffer. The analyte injection was repeated in ascending order of concentration for seven cycles. After each cycle, the sensor chip surface was completely regenerated using 10 mm Glycine‐HCl at a flow rate of 30 µL min^−1^ for 30 s to remove the analyte, followed by the next cycle.

### Enzyme Inhibition Assay

The enzymatic activity was performed by using nitrocefin. Briefly, 10^6^ CFUs mL^−1^ bacteria suspension was centrifuged, and the supernatant was divided into 1.5 mL centrifuge tubes. Next, different concentrations of GN1 were added into the supernatant and incubated for 30 min at 37 °C with 200 rpm. Nitrocefin (1 mg mL^−1^) was added to the mixture and if the color change to red was seen within 30 min, it indicated there is *β*‐lactamase activity. Then, 200 µL of the mixture was then aspirated in a 96‐well plate and the absorbance was measured at 380 nm (Tecan Infinite E Plex).

### Biofilm Inhibition Assay

Prevention of biofilm formation was assessed as described previously.^[^ ^46^ ^]^ Briefly, 10^6^ CFUs mL^−1^ bacteria suspension was incubated with drugs for 36 h at 37 °C in a humidified atmosphere. After incubation, planktonic bacteria were removed, and biofilms were stained with 0.1% crystal violet for 15 min, then washed, and solubilized with 33% acetic acid. The optical density at 570 nm was determined as a measure of biofilm mass.

### Membrane Potential Assay

MRSA membrane potential was examined by detecting the changes of fluorescent dye, DiOC_2_(3), using a flow cytometer.^[^ ^53^ ^]^ Decreased fluorescence intensity of DiOC_2_(3) indicates dissipated membrane potential, accompanied by a shift from red to green fluorescence. The membrane‐potential disrupter CCCP served as a positive control.

### Membrane Permeability Assay

The fluorescent dye PI assay was used to determine the cell membrane permeability. Briefly, PI was incubated with bacterial suspension for 30 min, subsequently, incubated with GN1 at 37 °C for 1 h. Then PI (0.5 µm) with an excitation wavelength of 535 nm and emission wavelength of 615 nm was measured using an Infinite E Plex Microplate reader (Tecan).

### Membrane Fluidity Assay

The exponential bacterial suspension was centrifuged and washed with PBS, then bacterial suspension was incubated with 10 µm Laurdan in dark for 30 min at 37 °C. The stained cell culture was washed with PBS twice times and concentrated. Next, different concentrations of GN1 were mixed with the stained bacterial cells to incubate for another 1 h at 37 °C. After 1 h incubation in the dark, the Laurdan fluorescence intensities were measured using a Microplate reader (Tecan, Männedorf, Switzerland) with emission wavelengths of 435 and 490 nm upon excitation at 350 nm. The Laurdan GP was calculated using the formula GP = (I435– I490) / (I435 + I490).

### Cellular ATP Level Assay

Intracellular ATP levels of MRSA T144 were determined using an Enhanced ATP Assay Kit (Beyotime, Shanghai, China). Briefly, the exponential bacterial suspension was centrifuged and washed with PBS, then incubated with different concentrations of GN1 for 1 h. After incubation, centrifuged and discard supernatant, ATP lysate was added to the bacterial cells. The ATP levels were monitored using Infinite E Plex Microplate reader (Tecan) in the model of luminescence.

### ROS Generation Measurement

2′,7′‐Dichlorodihydrofluorescein diacetate (DCFH‐DA, 10 µm) was applied to monitor the levels of ROS in MRSA T144. Briefly, bacteria were first incubated with DCFH‐DA (10 µm), then the probed cells were mixed with GN1 for 60 min. The ROS levels were assessed by monitoring the fluorescence intensity (λexcitation = 488 nm, λemission = 525 nm) using a Microplate reader (Tecan, Männedorf, Switzerland).

### NAC Assay

*N*‐acetyl‐L‐cysteine (NAC) was applied to evaluate the roles of ROS in the activity of GN1.^[^ ^20^ ^]^ Briefly, NAC (1, 5 and 10 mM) was added to the mixture of GN1 and bacteria culture. The effects of different concentrations of NAC on bacterial growth were assessed by measuring the OD_600_.

### Galleria Mellonella Infection Model

*Galleria mellonella* larvae (supplied by Huiyude Biotech Company, Tianjin, China) were separated into four groups (n = 12 per group) and infected by injecting MRSA T144 (10 µL, 1.0 × 10^6^ CFUs per larvae) into the right posterior gastropoda. The infected larvae were treated with GN1 (60 mg kg^−1^), PIP (50 mg kg^−1^) or a mixture of GN1 with PIP (60 + 50 mg kg) in the left posterior gastropoda after 1 h infection, with the PBS serving as a control treatment. The survival rates of *Galleria mellonella* larvae were recorded within 24 h.

### Rats Skin Wounds Infection Model

The therapeutic effect of GN1‐PIP combination against a clinical isolate of MRSA T144 was assayed in a wounded rat skin infection model. Briefly, 250 g experimental ICR male rats were anesthetized with pentobarbital (30 mg kg‐1) by intraperitoneal injection and were divided into four groups: PBS, GN1 (5 mg kg^−1^), PIP (25 mg kg^−1^) and GN1 + PIP combination (5 mg kg^−1^ + 25 mg kg^−1^). Rats were performed with wounds (1cm^2^) on the back and infected with 0.1 mL of bacterial suspension (1.0 × 10^7^ CFUs per wound). At 1 h post‐infection, PBS, GN1 (5 mg kg^−1^), PIP (25 mg kg^−1^) and GN1 + PIP combination (5 mg kg^−1^ + 25 mg kg^−1^) was administered to the wounds, lasting for 3 days of administration (once a day). Wound size was tested after treatments at the indicated times (0, 3, 5, 6, and 7 days). On the 7^th^ day, wound tissues were homogenized in 1 mL of sterile PBS and plated serial dilutions of the suspension on LB agar plates and incubating for 12 h. Moreover, some of wound tissue samples were excised and fixed in 10% formalin saline for 48 h. The samples were dewaxed in xylene and dehydrated in serial dilutions of alcohol and then processed in paraffin. These samples were then cut at 4 µm thicknesses for a traditional hematoxylin‐eosin (HE) and masson's trichrome staining. Additionally, different types of pro‐inflammatory mediators were determined in the serum by ELISA kits (Beyotime, Jiangsu, China).

### Statistics and Reproducibility

Statistical analysis was performed using GraphPad Prism version 9.0. All experiments were performed with biological replicates and data were shown as mean ± SD. Unpaired *t* test between two groups or one‐way analysis of variance (ANOVA) among multiple groups were used to calculate *P* values (*P < 0.05, **P < 0.01, ***P < 0.001, and ****P < 0.0001).

## Conflict of Interest

The authors declare no conflict of interest.

## Author Contributions

J.S. and C.C. contributed equally to the work. Y.L. and Z.W. designed and supervised the project. J.S. and C.C. performed experiments, analyzed data, and drafted the manuscript. P.K., F.Y., and Q.L. helped perform experiments. Y.L. and J.S. wrote and revised the manuscript. All the authors read and approved the final manuscript.

## Supporting information



Supporting Information

## Data Availability

The data that support the findings of this study are available from the corresponding author upon reasonable request.
