# Supplementary material for: Non‐Membrane Active Peptide Resensitizes MRSA to β‐Lactam Antibiotics and Inhibits S. aureus Virulence
Source: Adv Sci (Weinh). 2025 Feb 20;12(15):2416260. doi: 10.1002/advs.202416260 (PMC12005801; doi:10.1002/advs.202416260)
Supplement: Supplementary file 1 — Supporting Information [file ADVS-12-2416260-s001.docx]

**Supporting Information**

**Table S1 AMPs used in this study and their information, related to Figure 1A.**

| **AMPs** | **Sequence (N→C)** | **MIC^a^** | **MIC^b^** | **Potentiation (fold)^c^** | **Ref.** |
| --- | --- | --- | --- | --- | --- |
| Pleurocidin-a | GWGSFFKKAAHVGKHVGKAALTHYL-NH_2_ | 2 | 512 | 1 | ^[1]^ |
| WR-6 | WRWRWR-NH_2_ | 16 | 256 | 2 |  |
| WK-12 | Ac-WKVKWKVKWKVK-NH­_2_ | 32 | 128 | 4 | ^[2]^ |
| Mast-49 | FPILINLKALAALAKKIL-NH_2_ | 8 | 128 | 4 |  |
| Mast-MO | FLPIIINLKALAALAKKIL-NH_2_ | 2 | 512 | 1 | ^[3]^ |
| Mast-15 | IFLPIINLKALAALAKKIL-NH_2_ | 2 | 512 | 1 |  |
| GN1 | AKRIRKLIKKIFKKI | 16 | 4 | 128 | ^[4]^ |
| GP1 | RRWKWRRKIKKWL | 64/128 | >512 | 1 |  |
| YI12 | YLRLIRYMAKMI-NH­­_2_ | 2 | >512 | 1 | ^[5]^ |
| FK13 | FPLTWLKWWKWKK-NH_2_ | 8 | >512 | 1 |  |
| P2 | RGLGLALVRRPRGLGLALVRR | >128 | >512 | 1 | ^[6]^ |
| P33 | RLGIAVALRRPRLGIAVALRR | 64 | >512 | 1 |  |
| P5 | RALLTTLLRRPRALLTTLLRR | 64 | >512 | 1 |  |
| WFK-PG | RWFKFPGRWFKF-NH_2_ | 16 | 16 | 32 |  |
| KW-6 | KWKWKW-NH_2_ | >128 | >512 | 1 |  |
| NAP-G_1_D-11 | Nap-GFFYALLSRVD | >128 | >512 | 1 |  |
| GL-13-2 | GLSLLLSLGLKLL | >128 | >512 | 1 |  |
| GL-13 | GLLSLLSLLGKLL-NH_2_ | 16 | 128 | 4 |  |
| IF-5 | Ile-Met-Ile-Ser-Phe | >128 | >512 | 1 |  |
| SNGD | SNGDGTLDAGSTCAPFYARA | >128 | >512 | 1 |  |
| P1 | RLVRILVSKRPVAIKPYFRL | >128 | 64 | 8 | ^[7]^ |
| Cathelicidin-BF15 | VKRWKKWKRKWKKWV-NH_2_ | 16 | 64 | 8 | ^[8]^ |
| CE-11 | Ac-CLPAFEGRNCE-NH_2_ | >128 | >512 | 1 |  |
| ND-9 | NRWCFAGDD | >128 | >512 | 1 | ^[9]^ |
| SF-12 | Ac-SCKDQLQSYICF-NH_2_ | >128 | >512 | 1 |  |
| WW291 | WWWLRKIW-NH_2_ | 8 | 256 | 2 | ^[10]^ |
| WW304 | WWWLRRRW-NH_2_ | 16 | >512 | 1 |  |
| AD-7 | ALLSRVD | >128 | >512 | 1 |  |

MIC^a^, MIC in the presence of AMPs only; MIC^b^, MIC of piperacillin in the presence of 1/4 MIC AMPs;

Potentiation (fold)^c^, MIC^c^ / MIC^b^ (MIC^c^, MIC^c^ in the presence of piperacillin only).

**Table S2 Potentiation activity of GN1 with different classes of β-lactams against MRSA T144, related to Figure 1B.**

| **Antibiotics** | **MIC^a^ (μg/mL)** | **MIC^b^ (μg/mL)** | **Potentiation (fold)^c^** |
| --- | --- | --- | --- |
| Ampicillin | 512 | 8 | 64 |
| Piperacillin | >512 | 8 | 64 |
| Carbenicillin | 32 | 1 | 32 |
| Ticarcillin | 16 | 1 | 16 |
| Oxacillin | 1 | 0.03125 | 32 |
| Ceftriaxone | 32 | 4 | 8 |
| Cefuroxime | 8 | 1 | 8 |
| Cefepime | 8 | 2 | 4 |
| Meropenem | 1 | 0.0625 | 16 |

MIC^a^, MIC in the presence of antibiotics only;

MIC^b^, MIC in combination of antibiotics and 1/4 MIC GN1;

Potentiation (fold)^c^, MIC^a^ / MIC^b^

**Table S3 Thermal, pH, salts and protease stability of GN1 against MRSA T144.**

| **Treatments** | | | | | | | | |
| --- | --- | --- | --- | --- | --- | --- | --- | --- |
| **Temperature** | **20℃** | | **40℃** | **60℃** | | **80℃** | **100℃** | **121℃** |
| MIC | 16 | | 16 | 16 | | 16 | 16 | 16 |
| **pH** | **2** | | **4** | **6** | | **8** | **10** | **12** |
| MIC | 16 | | 16 | 16 | | 16 | 16 | 16 |
| **Salt ions (10 mM)** | **Na^+^** | **K^+^** | | **Mg^2+^** | **Ca^2+^** | | **Fe^3+^** | |
| MIC | 32 | 32 | | 64 | >128 | | >128 | |
| **Protease (1 mg/mL)** | **Pepsin** | | | **Trypsin** | | | **Papain** | |
| MIC | 128 | | | 128 | | | >128 | |
|  | **Serum (10%)** | | | **DMEM (10%)** | | |  | |
| MIC | 4 | | | 16 | | |  | |

**Table S4 Thermal, pH, salts and protease stability of D-GN1 against MRSA T144.**

| **Treatments** | | | | | | | | |
| --- | --- | --- | --- | --- | --- | --- | --- | --- |
| **Temperature** | **20℃** | | **40℃** | **60℃** | | **80℃** | **100℃** | **121℃** |
| MIC | 16 | | 16 | 16 | | 16 | 16 | 16 |
| **pH** | **2** | | **4** | **6** | | **8** | **10** | **12** |
| MIC | 16 | | 16 | 16 | | 16 | 16 | 16 |
| **Salt ions (10 mM)** | **Na^+^** | **K^+^** | | **Mg^2+^** | **Ca^2+^** | | **Fe^3+^** | |
| MIC | 32 | 32 | | >128 | >128 | | >128 | |
| **Protease (1 mg/mL)** | **Pepsin** | | | **Trypsin** | | | **Papain** | |
| MIC | 32 | | | 32 | | | 32 | |
|  | **Serum (10%)** | | | **DMEM (10%)** | | |  | |
| MIC | 8 | | | 8 | | |  | |

**Table S5 MIC of different β-lactams with/without GN1 against *S. aureus* 29213.**

| Antibiotics | MIC (μg/mL) |
| --- | --- |
| AMP MIC | 16 |
| AMP MIC (+1/2 MIC GN1) | 8 |
| PIP MIC | 2 |
| PIP MIC (+1/2 MIC GN1) | 0.5 |
| TIC MIC | 2 |
| TIC MIC (+1/2 MIC GN1) | 2 |
| SRB MIC | 8 |
| SRB MIC (+1/2 MIC GN1) | 4 |
| MEM MIC | 0.0625 |
| MEM MIC (+1/2 MIC GN1) | 0.03125 |
| FEP MIC | 4 |
| FEP MIC (+1/2 MIC GN1) | 2 |

**Table S6 Affinity of PBP2a-GN1 molecular docking.**

| **Modes** | **Affinity (kcal/mol)** |
| --- | --- |
| PBP2a-GN1 01 | -5.3 |
| PBP2a-GN1 02 | -4.9 |
| PBP2a-GN1 03 | -4.9 |
| PBP2a-GN1 04 | -4.8 |
| PBP2a-GN1 05 | -4.8 |
| PBP2a-GN1 06 | -4.8 |
| PBP2a-GN1 07 | -4.7 |
| PBP2a-GN1 08 | -4.7 |
| PBP2a-GN1 09 | -4.7 |

**Table S7 Affinity of β-lactamase-GN1 molecular docking.**

| **Modes** | **Affinity (kcal/mol)** |
| --- | --- |
| β-lactamase -GN1 01 | -4.2 |
| β-lactamase -GN1 02 | -4.1 |
| β-lactamase -GN1 03 | -4.1 |
| β-lactamase -GN1 04 | -3.9 |
| β-lactamase -GN1 05 | -3.9 |
| β-lactamase -GN1 06 | -3.8 |
| β-lactamase -GN1 07 | -3.7 |
| β-lactamase -GN1 08 | -3.6 |
| β-lactamase -GN1 09 | -3.6 |

**Figures**

**
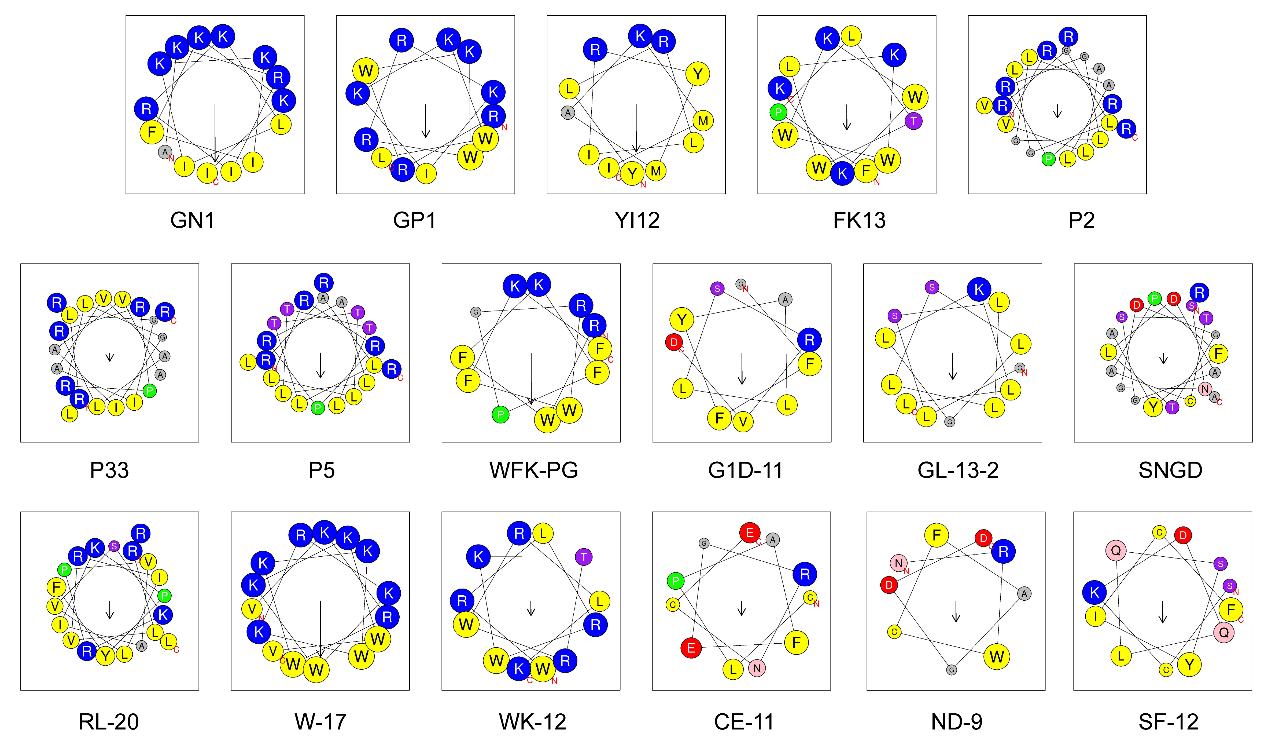
**

**Figure S1. Helical wheel projections of the screened AMP using HeliQuest analysis.**

Helical wheels of AMPs were presented using HeliQuest analysis (http://heliquest.ipmc.cnrs.fr/cgi-bin/ComputParamsV2.py).


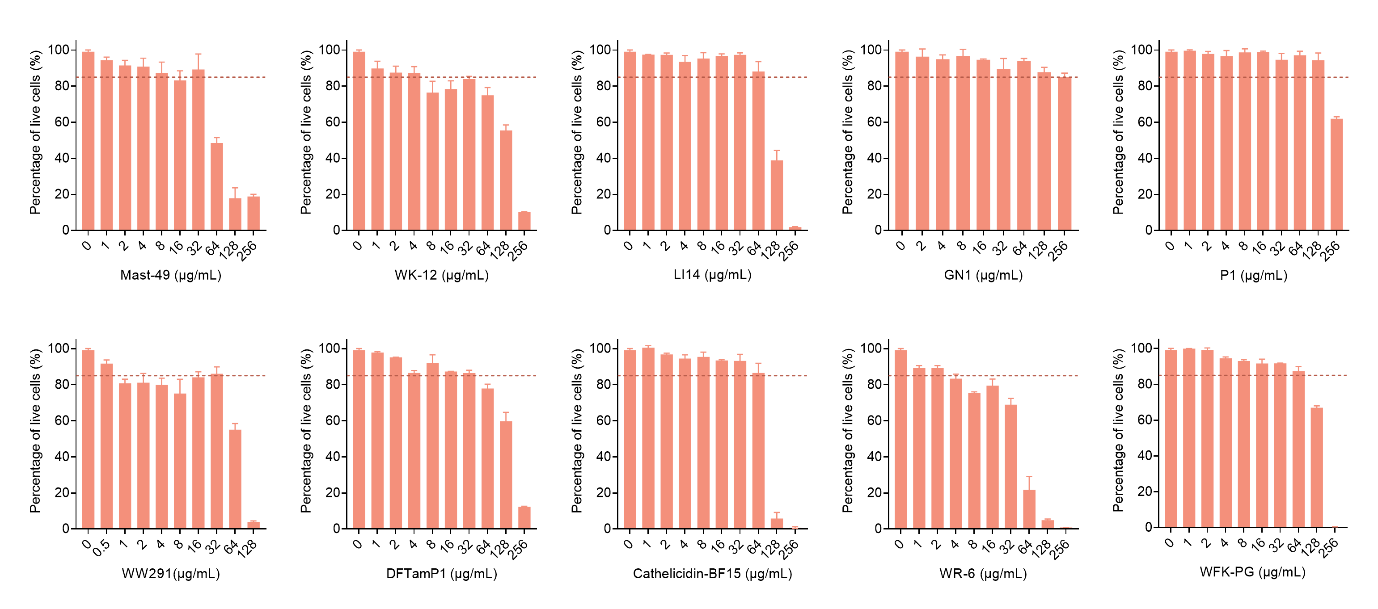


**Figure S2. Cytotoxicity analysis of AMPs in RAW264.7 cells.**

Cytotoxicity of selected AMPs to macrophage RAW264.7 cells at various concentrations was evaluated by calculating cell survival rate. All experiments were conducted with three biological replicates and data were presented as mean ± SD.


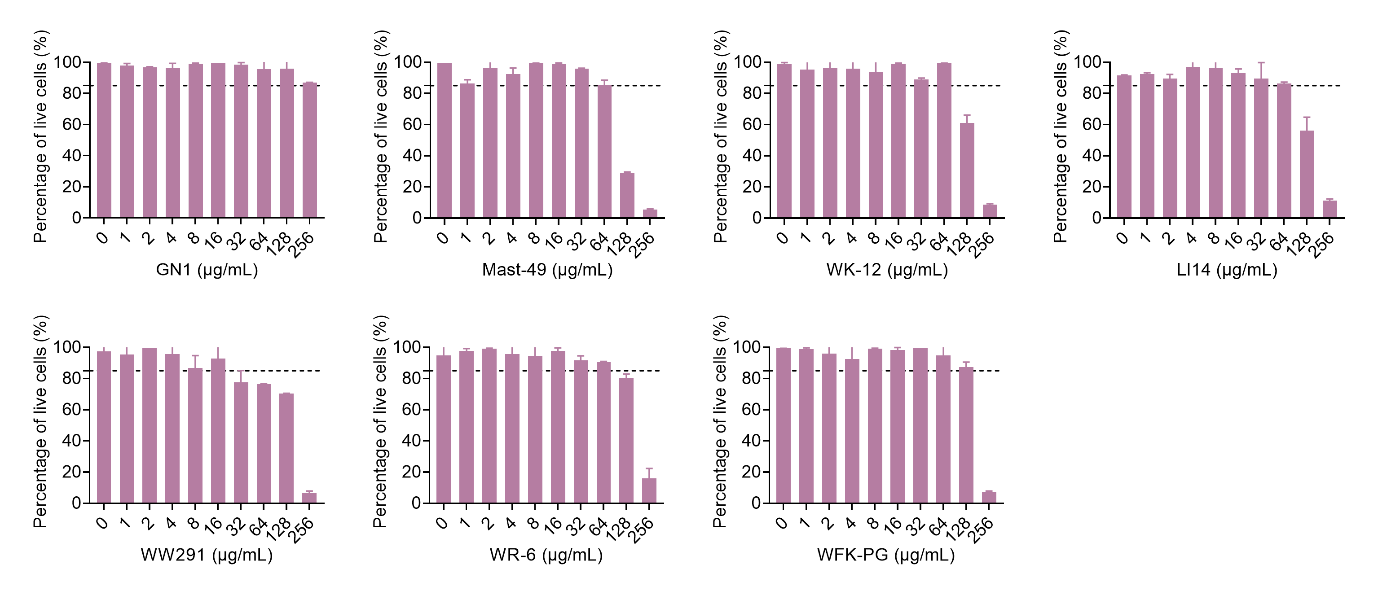


**Figure S3. Cytotoxicity analysis of AMPs in HEK293T cells.**

Cytotoxicity of selected AMPs to HEK293T cells at various concentrations was evaluated by calculating cell survival rate. All experiments were conducted with three biological replicates and data were presented as mean ± SD.


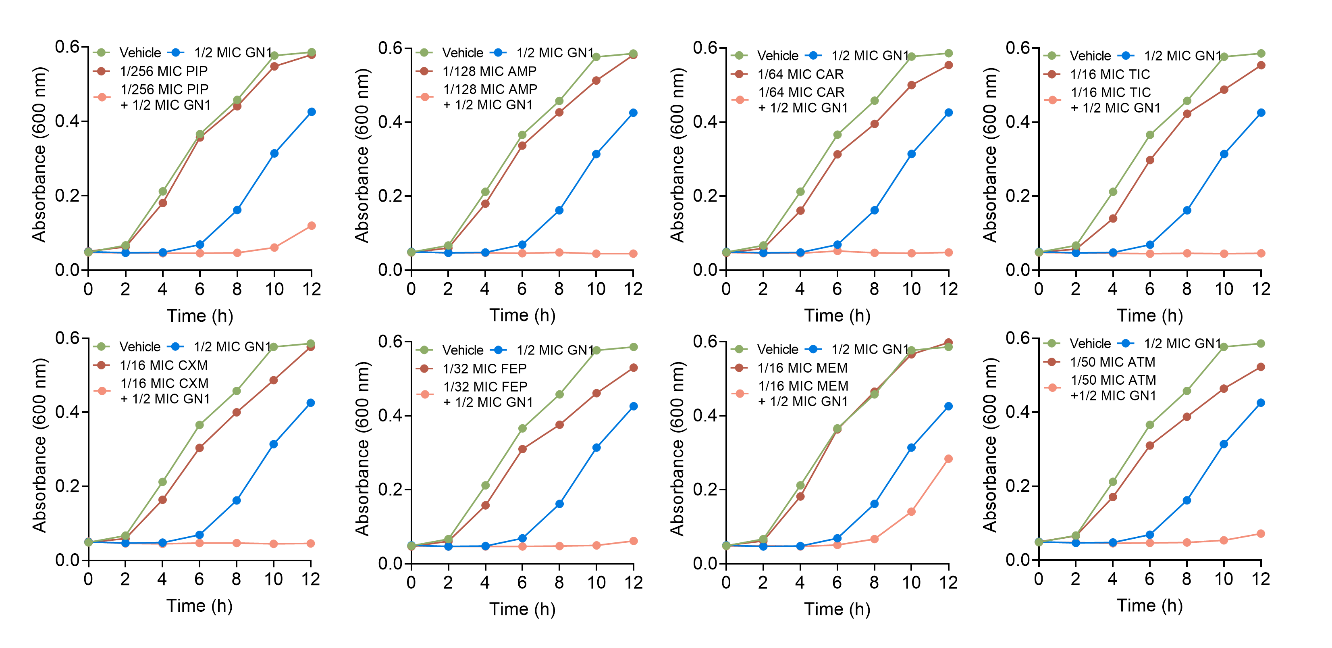


**Figure S4. Combinations of GN1 and β-lactams inhibit the growth of MRSA T144.**

The absorbance at 600 nm was measured during 12 h and incubation at 37℃. All experiments were conducted with three biological replicates and data were presented as mean ± SD.


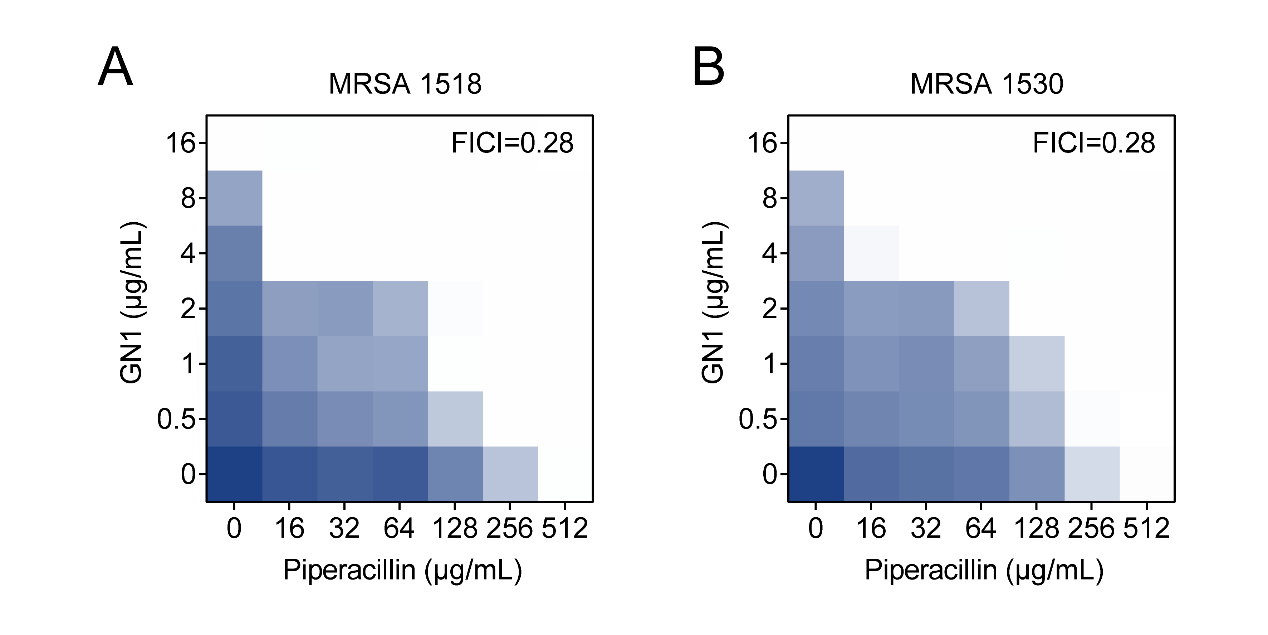


**Figure S5. Checkerboard assay of GN1 and PIP against MRSA 1518 and MRSA 1530.**

OD_600_ nm was measured after 18 h incubation at 37℃ and the fractional inhibitory concentration index (FICI) value was 0.28. Data represent the mean absorbance at 600 nm of biological replicates.


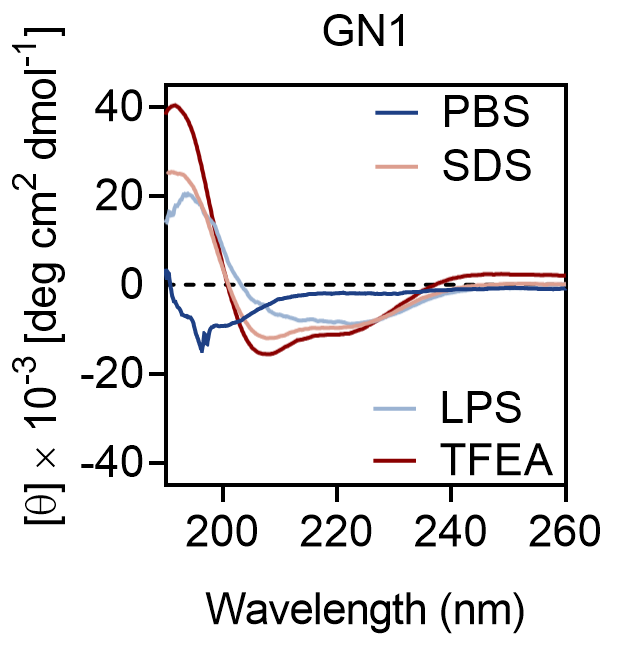

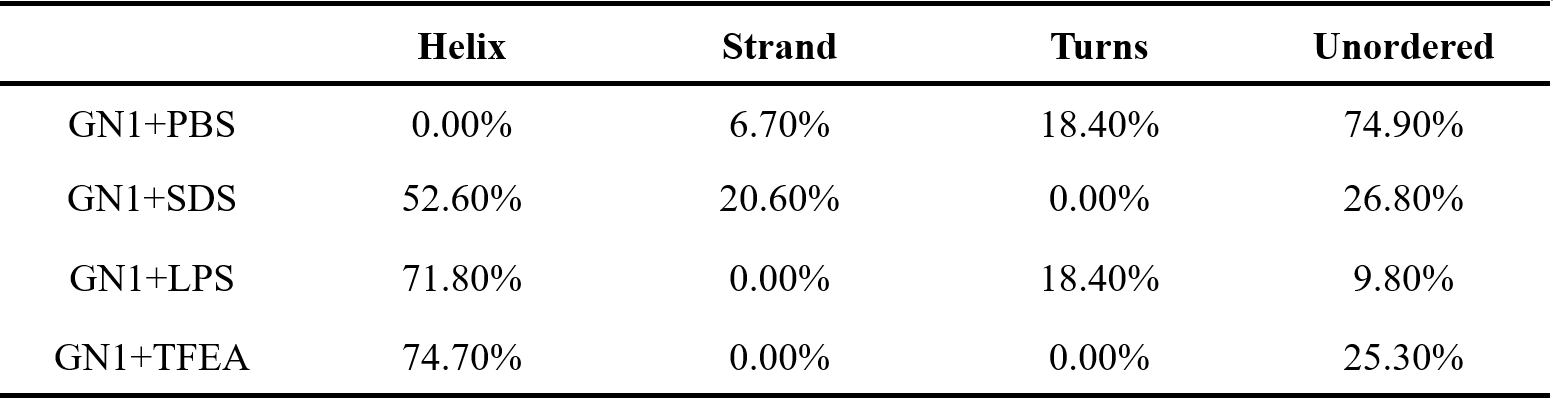


**Figure S6. Circular dichroism (CD) spectra of GN1 in various solutions.**

PBS (10 mM, pH = 7.4), lipopolysaccharide (LPS) (50 µM), sodium dodecyl sulfate (SDS) (50 mM), and 50% trifluoroethano (TFEA) were used. Experiments were conducted with three biological replicates and data were obtained from three independent scans per sample.


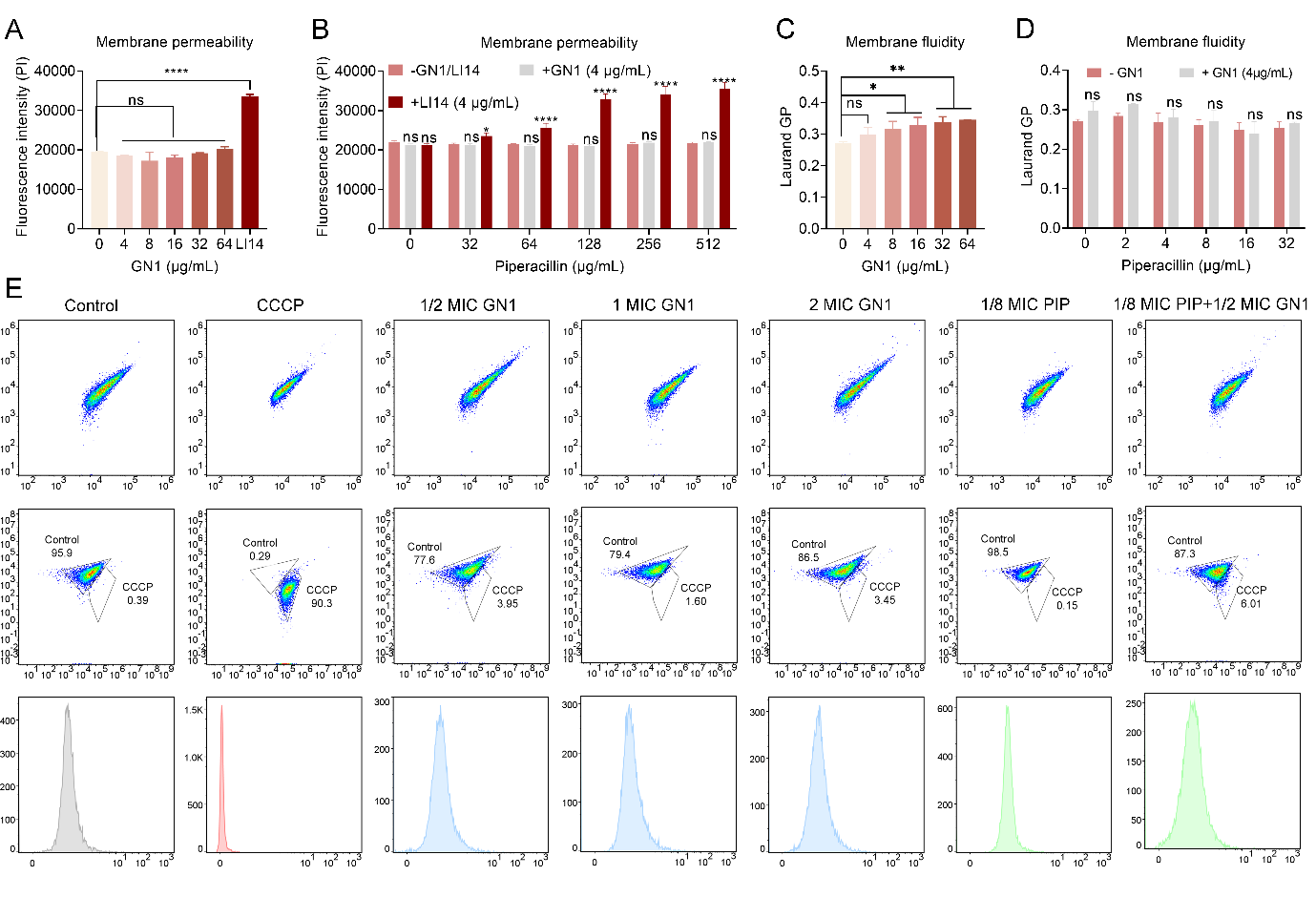


**Figure S7. GN1 did not disrupt bacterial membrane permeability and potential.**

**(A-B)** Membrane permeability of MRSA T144 treated with GN1/LI14 **(A)**, piperacillin alone or combination with GN1/LI14 **(B)**; Propidium iodide (PI) was used to measure membrane permeability, with an excitation/emission wavelength of 535 nm/615 nm. LI14, a membrane active peptide, was used as the positive control.

**(C-D)** Membrane fluidity under the addition of GN1 **(C)**, piperacillin alone or combination with GN1 **(D)**; Membrane fluidity was determined using 10 µM Laurdan, and the fluorescence intensities were detected with emission wavelengths of 435 nm and 490 nm upon excitation at 350 nm.

**(E)** Membrane potential was examined by using Flow cytometer. MRSA membrane potential was examined by detecting the changes of fluorescent dye, DiOC_2_(3), using a flow cytometer.

CCCP (carbonyl cyanide m-chlorophenylhydrazone), a membrane potential disruptor, was used as positive control.

All experiments were conducted with three biological replicates and data were presented as mean ± SD. Statistical significance was analyzed by ordinary one-way or two-way ANOVA (**P* < 0.05, ***P* < 0.01, *****P* < 0.0001). ns, not significant


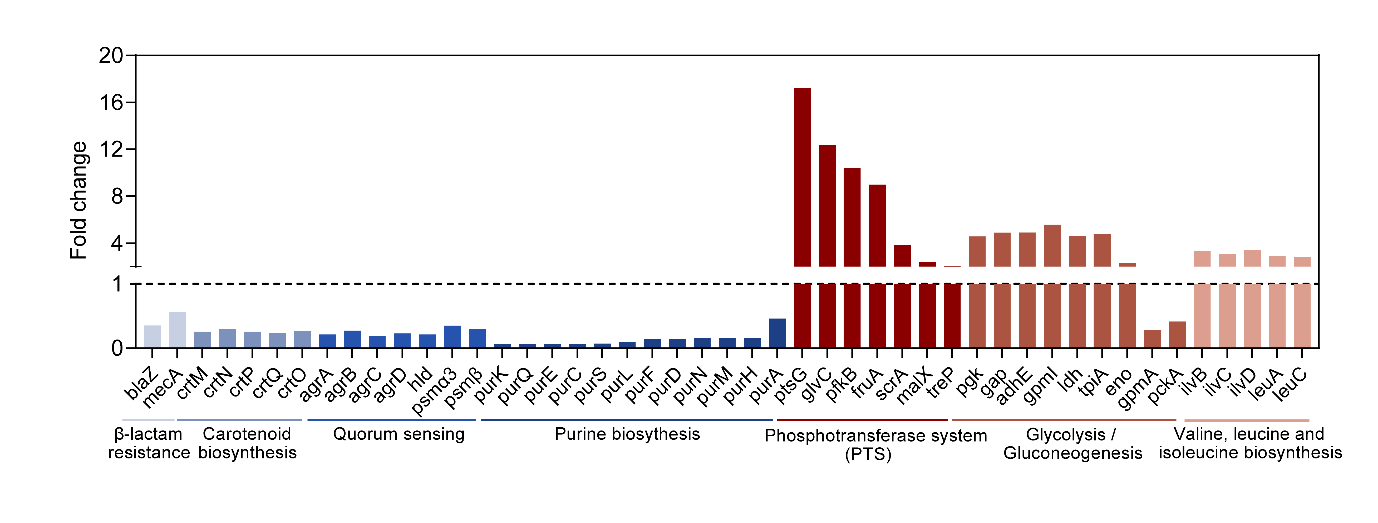


**Figure S8. Multiples of gene expression levels in different pathways under GN1 action.**

Fold changes of selected genes involved in β-lactamase resistance, carotenoid biosynthesis, quorum sensing, purine biosynthesis, phosphotransferase system, glycosis/gluconeogenesis and valine, leucine and isoleucine biosynthesis.


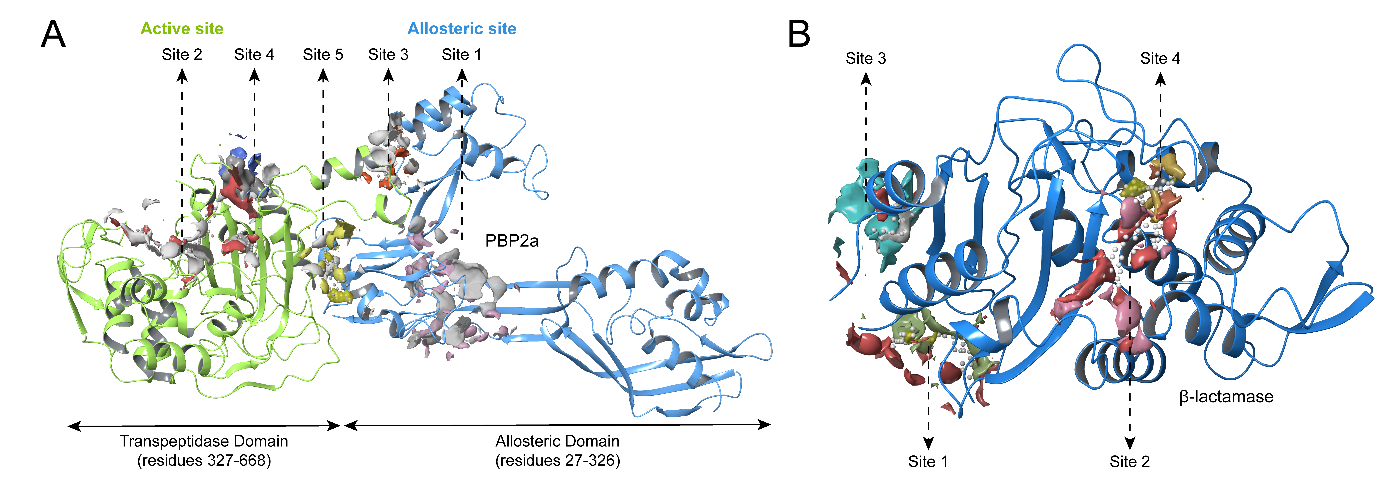


**Figure S9. The potential binding sites on PBP2a and β-lactamase.**

The first five potential binding sites on PBP2a (A) and four potential binding sites on β-lactamase (B) were identified by SiteMap, named site 1-5 and 1-4, respectively.


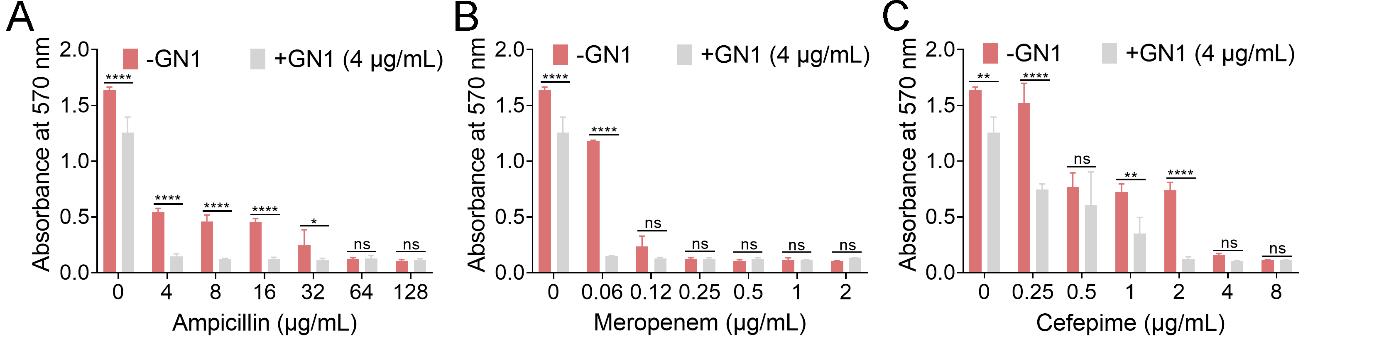


**Figure S10. GN1 potentiates the anti-biofilm ability of β-lactam antibiotics.**

**(A-C)** Bacterial biofilm formation of MRSA T144 under ampicillin (AMP), meropenem (MEM) and cefepime (FEP) in the absence/presence of 1/2 MIC GN1 against MRSA T144. Biofilm mass was quantified by measuring sample absorbance at 570 nm;

Experiments were conducted with three biological replicates and data were presented as mean ± SD. Statistical significance was analyzed by two-way ANOVA (**P* < 0.05, ***P* < 0.01, *****P* < 0.0001). ns, not significant.


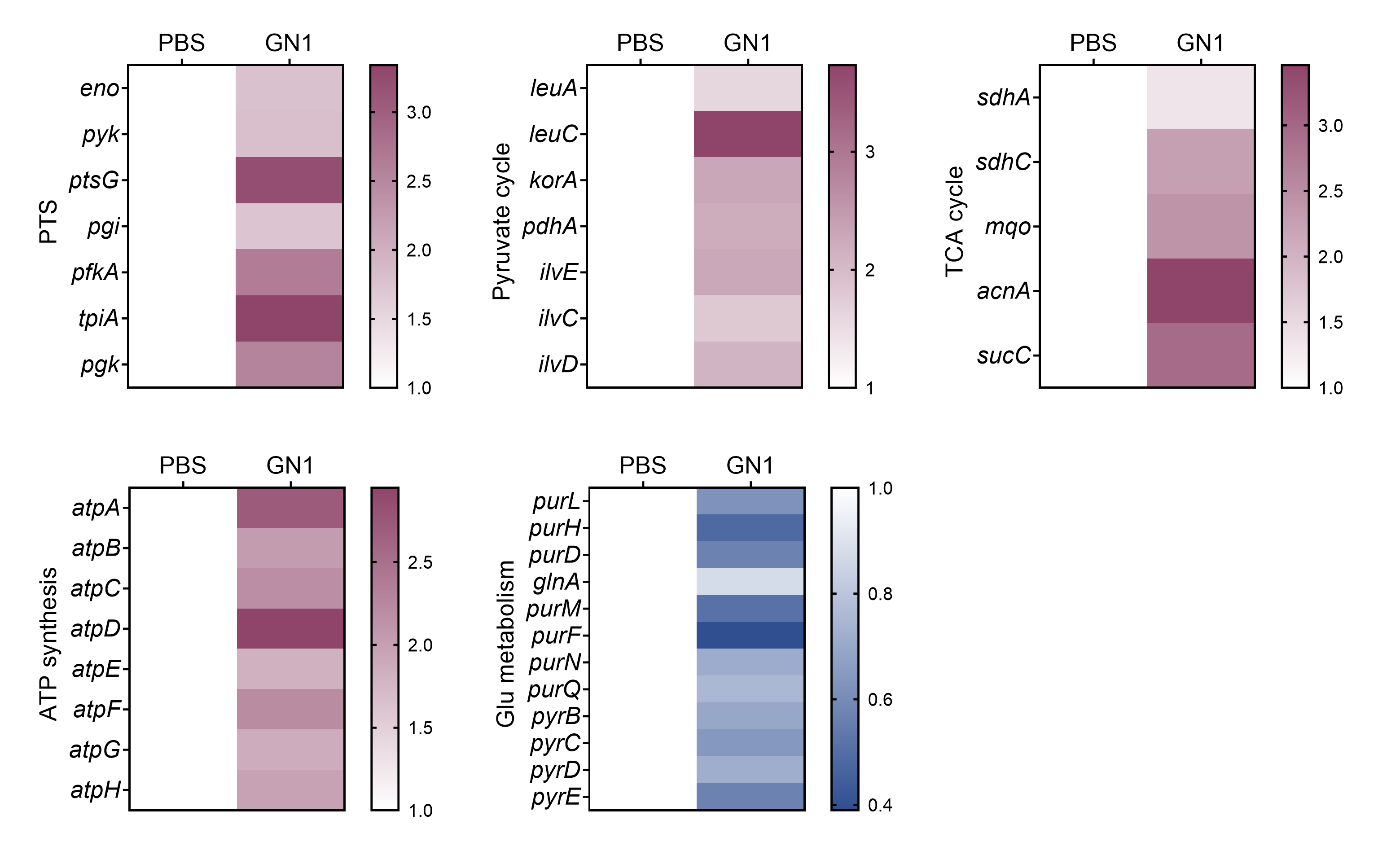


**Figure S11. RT-qPCR analysis of the expression of energy metabolism and glutamate metabolism-related genes after exposure to GN1.**


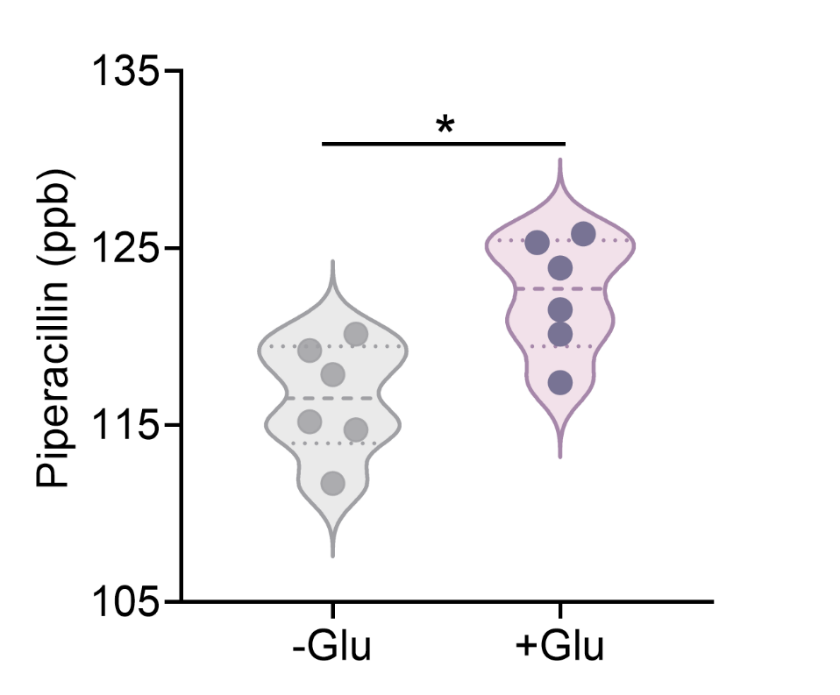


**Figure S12. Effect of exogenous glutamate addition on the intracellular accumulation of piperacillin in MRSA T144.**

Experiments were conducted with three biological replicates and data were presented as mean ± SD. Statistical significance was analyzed by unpaired *t* test (**P* < 0.05).

**References**

[1] H.-C. Hsu, M.-H. Chen, M.-L. Yeh, W.-J. Chen, *Marine Drugs* **2022**, *20*, 519.

[2] Y. Lyu, T. Chen, L. Shang, Y. Yang, Z. Li, J. Zhu, A. Shan, *J. Med. Chem.* **2019**, *62*, 6941.

[3] O. N. Silva, M. D. T. Torres, J. Cao, E. S. F. Alves, L. V. Rodrigues, J. M. Resende, L. M. Lião, W. F. Porto, I. C. M. Fensterseifer, T. K. Lu, et al., *Proc. Natl. Acad. Sci. U. S. A.* **2020**, *117*, 26936.

[4] A. Capecchi, X. Cai, H. Personne, T. Köhler, C. van Delden, J.-L. Reymond, *Chem. Sci.* **2021**, *12*, 9221.

[5] P. Das, T. Sercu, K. Wadhawan, I. Padhi, S. Gehrmann, F. Cipcigan, V. Chenthamarakshan, H. Strobelt, C. Dos Santos, P.-Y. Chen, et al., *Nat. Biomed. Eng.* **2021**, *5*, 613.

[6] G. Wu, L. Khodaparast, L. Khodaparast, M. De Vleeschouwer, J. Housmans, B. Houben, J. Schymkowitz, F. Rousseau, *Cell Chem. Biol.* **2021**, *28*, 524.

[7] A. T. Tucker, S. P. Leonard, C. D. DuBois, G. A. Knauf, A. L. Cunningham, C. O. Wilke, M. S. Trent, B. W. Davies, *Cell* **2018**, *172*, 618.

[8] J. Mwangi, Y. Yin, G. Wang, M. Yang, Y. Li, Z. Zhang, R. Lai, *Proc. Natl. Acad. Sci. U. S. A.* **2019**, *116*, 26516.

[9] M. L. Teixeira, A. D. Rosa, A. Brandelli, *Microbiology* **2013**, *159*, 980.

[10] J. Lakshmaiah Narayana, B. Mishra, T. Lushnikova, Q. Wu, Y. S. Chhonker, Y. Zhang, D. Zarena, E. S. Salnikov, X. Dang, F. Wang, et al., *Proc. Natl. Acad. Sci. U. S. A.* **2020**, *117*, 19446.
